# Safflower Extract Ameliorates Cisplatin-Induced Acute Kidney Injury by Regulating Microbiota-Metabolic-Redox Nexus and PI3K–Akt/Nrf2 Pathway

**DOI:** 10.3390/antiox15070855

**Published:** 2026-07-07

**Authors:** Yue Chang, Yanzhuo Song, Naveed Ahmad, Chao Song, Yuhang Chu, Yuru Zhang, Lufei Feng, Wei Wei, Min Zhang, Xiuming Liu

**Affiliations:** 1 College of Life Sciences, Engineering Research Center of the Chinese Ministry of Education for Bioreactor and Pharmaceutical Development, Jilin Agricultural University, Changchun 130118, China; cy18391348615@163.com (Y.C.);; 2 Institute for Safflower Industry Research of Shihezi University/Pharmacy College of Shihezi University/Key Laboratory of Xinjiang Phytomedicine Resource and Utilization, Ministry of Education, Shihezi 832003, China; naveed@shzu.edu.cn (N.A.);; 3 Ginseng and Antler Products Testing Center of the Ministry of Agriculture PRC, Jilin Agricultural University, Changchun 130118, China; 4 College of Animal Science and Technology, Jilin Agricultural Science and Technology College, Jilin 132101, China; weiwei@jlnku.edu.cn

**Keywords:** acute kidney injury, flavonoids, nephroprotection, multi-omics, microbial diversity

## Abstract

Cisplatin-induced acute kidney injury (AKI) remains a dose-limiting complication in cancer chemotherapy with restricted preventive measures. *Carthamus tinctorius* L. (safflower) is known to exhibit effective antioxidant and anti-inflammatory properties; however its potential in renoprotective mechanisms remains poorly understood. The present study utilized a cisplatin-induced AKI mouse model to evaluate the renoprotective potential of CT (*Carthamus tinctorius*) extract. Integrated multi-omics along with in silico and in vivo approaches were used to elucidate the underlying mechanisms of action. The results initially demonstrated a rich phytochemical profile of CT extract characterized by abundant polysaccharides and flavonoids, with Hydroxysafflor Yellow A as a dominant bioactive constituent. In a cisplatin-induced acute kidney injury (AKI) mouse model, CT extract noticeably ameliorated the abnormalities of renal injury, as suggested by improved histopathology, reduced serum creatinine and BUN levels, and regulation of redox homeostasis. Metabolically, CT extract partially reversed AKI-associated disturbances by affecting 21 key metabolites, likely associated with histidine and alanine-aspartate-glutamate biosynthesis, and modulating amino acid and energy metabolism pathways. Concurrently, CT extract improved gut microbial homeostasis, increasing microbial diversity, normalizing the Firmicutes/Bacteroidota ratio, suppressing pathogens, and enriching beneficial Ligilactobacillus. Network pharmacology and molecular docking identified AKT1, RELA, MAPK, and TP53 as central targets of core compounds (rutin and kaempferol derivatives), apparently targeting the PI3K-AKT and RELA (NF-kappaB) hubs. These results suggested that the renoprotective effects of CT extract are associated with transcriptional upregulation of the *PI3K/Akt/Nrf2* pathway-related genes, increased expression of antioxidant genes (*Ho-1*, *Sod1*), and reduced expression of pro-inflammatory mediators (*RelA*, *Cdk2*) in the cisplatin-induced AKI mouse model.

## 1. Introduction

Cisplatin-induced nephrotoxicity remains a significant clinical hurdle, affecting roughly one-third of patients and creates a major bottleneck in achieving optimal dosing for cancer patients [1,2]. CDDP-induced AKI is driven by a self-amplifying cycle of tubular epithelial apoptosis, mitochondrial dysfunction, oxidative stress, and inflammatory cascades [3,4,5,6]. Existing prophylactic approaches, such as hyperhydration, diuresis, and antioxidant supplementation provide only modest renal protection while risking electrolyte imbalance and reduced antitumor potency [7,8,9]. Consequently, there is an urgent, unmet need for safe, multitargeted renoprotective agents that can prevent cisplatin-induced renal injury without compromising its chemotherapeutic efficacy.

Given the systemic nature of cisplatin-induced injury, exploring deep-rooted ethnobotanical prospects for identifying agents capable of mitigating drug-induced toxicity presents a rational framework. *Carthamus tinctorius* L. (safflower) recognized for its well-known pharmacological landscape. Its traditional application in regulating blood circulation and the resolution of stasis to treat trauma and amenorrhea [10]. Notably, safflower is a primary bioactive component in classical formulations such as the Xuefu Zhuyu (XFZY) and Taohong Siwu (THSWD) decoctions. The therapeutic scope of safflower extends to compound formulas aimed at alleviating edema, suggesting a potential relevance to fluid metabolism and renal function. For instance, safflower is a key component in several TCM formulations used for renal conditions, such as Xuefu Zhuyu decoction (XFZY) and Taohong Siwu Decoction (THSWD), which are clinically applied to treat chronic kidney disease and diabetic nephropathy by promoting blood circulation and reducing stasis [11,12]. Safflower seeds has been shown to significantly attenuated renal dysfunction and pathological damage in a mouse model of cisplatin-induced nephrotoxicity [13].

Safflower-derived quinochalcone Hydroxysafflor Yellow A (HSYA) has been previously known to serve as a renoprotective agent to counter ischemia–reperfusion injury via Akt/Nrf2-signaling [14]. HSYA has also been shown to suppress the TLR4/NF-κB axis, exhibiting protective effects against renal ischemia–reperfusion [15]. Other key flavonoids including rutin and quercetin have emerged as independent bioactive substances to reduce renal injury by activating anti-ferroptosis and macrophage polarization cascades [16,17]. Although current studies offer important insights into safflower-mediated nephroprotection mechanisms, these have largely focused on individual compounds. However, a whole plant extract may offer synergistic advantages over single purified compounds, as multiple constituents can concurrently modulate several pathophysiological pathways (e.g., oxidative stress, inflammation, and gut microbiota) through multi-target mechanisms, potentially achieving superior efficacy with lower toxicity. Therefore, the effects of a standard safflower extract (CT extract) in mitigating cisplatin-induced nephrotoxicity has not been previously explored from this integrative perspective. In addition, it also remains unclear whether CT extract can act beyond direct cellular mechanisms by reshaping the gut–kidney metabolic axis, which is increasingly being recognized as a major driver of AKI progression.

To address these important knowledge gaps, we investigated the protective efficiency of a chemically characterized CT extract in mitigating cisplatin-induced nephrotoxicity. We employed an integrated multi-omics approach to uncover the systemic immunometabolic modulation nexus using a murine model of cisplatin-induced AKI. Further validation was performed with histopathological assessment and molecular characterization. Our research provides a scalable multi-omics resource for establishing safflower-derived adjunct remedies guided by protective effects of specialized plant metabolites in complex disease models.

## 2. Materials and Methods

### 2.1. Preparation of CT Extract

Safflower petals were obtained from the Tacheng Prefecture, Xinjiang Uygur Autonomous Region, China. To confirm the identity of purchased material of *Carthamus tinctorius* L. (safflower), several morphological traits against the verified voucher specimen (KUN: 1449300; Collection No: 16CS13094, accessible through the Chinese Virtual Herbarium) were carried out. The petals were pulverized using a grinding mill and sieved through a 100-mesh sieve to obtain homogeneous powder. The powder was mixed with 80% ethanol at a solid-to-solvent ratio of 1:20 (*w*/*v*). Ultrasonic-assisted extraction was performed using a JY99-IIDN ultrasonic cell disruptor (Ningbo Scientz Biotechnology Co., Ltd., Ningbo, China) under the following parameters: 400 W power output, 120 min total duration. The extract was lyophilized to yield the final powder, which was sealed and stored at −80 °C prior to use.

### 2.2. Quantification of Bioactive Compounds from CT Extract Using LC-MS Analysis

The quantification of Acacetin, Luteolin, Astragalin, Isoquercitrin, Kaempferol-3-O-rutinoside, Protocatechuic acid, Quercetin, Rutin, Taxifolin, Hydroxysafflor yellow A, Kaempferol, Apigenin, and Naringenin in CT extract was performed using LC-MS analysis. A series of standard operating solutions of different concentrations were obtained by diluting the mixed standard stock solutions. Chromatographic separation was performed on a Phenomenex Kinetex column (2.1 × 100 mm, 2.6 µm) with an injection volume of 1 µL, using a Prominence LC-40 system (Shimadzu Corporation, Kyoto, Japan) coupled to a SCIEX Triple Quad™ 7500 mass spectrometer (SCIEX, Framingham, MA, USA). The mobile phase consisted of (A) methanol and (B) 0.1% formic acid in water, operated at a flow rate of 0.3 mL/min. The gradient elution program was as follows: 0–1 min, 90% B; 7 min, 80% B; 15 min, 40% B; 16 min, 10% B; 17 min, 10% B; 17.1–20 min, 90% B. All sample solutions were filtered through a 0.45 µm membrane filter prior to analysis. The polysaccharide content in the CT extract was determined using the phenol-sulfuric acid method.

### 2.3. Animal Experimental Design and Sample Collection

Approximately 6-week-old male C57BL/6J mice were randomly divided into three groups (*n* = 8): Control (Con), acute kidney injury model (AKI), and AKI+CT extract intervention (AKI+CT extract). All mice received the assigned treatments as described below. No animal was excluded from the experiment due to death or illness; all eight mice in each group completed the entire in vivo protocol.

The AKI+CT extract group received CT extract (40 mg/kg, the dose of 40 mg/kg was determined based on preliminary dose-finding experiments, dissolved in saline) via oral feeding daily for 16 consecutive days starting from day 1, while the Con and AKI groups received an equal volume of saline. On day 14 (3 h after feeding), the AKI and AKI+CT extract groups were intraperitoneally injected with a single dose of cisplatin (20 mg/kg, dissolved in saline; Macklin D807330) to induce acute kidney injury, and the Con group received an equivalent volume of saline. All mice were euthanized 72 h after cisplatin injection (i.e., day 17) by CO_2_ asphyxiation.

Blood, urine, kidney tissues, and cecal contents were collected from all animals. One kidney was fixed in 4% paraformaldehyde for H&E staining; the other was snap-frozen for biochemical and molecular analyses.

For the assays presented in this study (serum biochemistry, renal oxidative stress markers, qPCR), the final sample size was n = 6 per group. This reduction from n = 8 to n = 6 occurred exclusively at the post-mortem sample processing stage and followed a two-stage, group-balanced procedure, rather than a single random draw from the entire 24-animal pool. First, because the serum and tissue matrices differ among the three groups (healthy, injured, and treated), we intentionally selected one animal from each group (3 animals in total) to establish the optimal assay conditions that would be valid across all experimental groups. This was a deliberate experimental design, not a random loss, and it reduced all three groups synchronously from n = 8 to n = 7. Second, during RNA quality assessment, exactly one sample per group failed to meet the predefined criteria (A260/A280 ratio outside 1.8–2.0, or evidence of degradation) and was excluded. Since all animals were subjected to identical housing, sampling, and storage procedures, the quality failure rate was evenly distributed across groups, resulting in a synchronous drop from n = 7 to n = 6 in every group. The specific samples that failed were random within each group, but the number of exclusions was equal across groups. Therefore, the equal final sample sizes (n = 6 per group) are not a coincidence of random chance, but rather the consequence of a balanced experimental design combined with uniformly distributed quality failures. No living animal was used for any purpose outside the approved experimental protocol.

The experimental protocol, which included the use of 8 mice per group for all treatments and analyses described herein, was approved by the Animal Care and Use Ethics Committee of Jilin Agricultural Science and Technology College (Approval No. LLSC202502030).

### 2.4. Serum and Hepatic Biochemical Analyses

Whole blood samples were centrifuged to obtain serum, and the activities of creatinine (CRE) and urea nitrogen (BUN) were determined using commercial assay kits (Nanjing Jiancheng Institute of Bioengineering, Nanjing, China) according to the manufacturer’s protocols. For renal oxidative stress analysis, kidney tissues were homogenized in pre-cooled saline buffer and centrifuged (2500 rpm, 15 min, 4 °C). The final supernatant was then tested for Malondialdehyde (MDA), Superoxide Dismutase (SOD), reduced Glutathione (GSH), and Catalase (CAT) activities, following the instructions given by standardized kits.

### 2.5. Histological Analysis of Renal Tissue

The specimens obtained from the kidney tissue were stabilized in 4% paraformaldehyde (PFA) using the phosphate-buffered saline solution, dehydrated, and embedded in paraffin blocks. Then, approximately 5-μm sections were prepared using a Leica RM2235 rotary microtome (Leica Microsystems, Wetzlar, Germany). For the histopathological assessment, hematoxylin and eosin (H&E) staining was carried out with reagents from Servicebio (Wuhan, China), and then followed by microscopic evaluation under an Olympus CX23 light microscope (Olympus Corporation, Tokyo, Japan).

### 2.6. Urine Metabolome Analysis Using LC-MS

An equal amount of 100 μL urine samples were taken and subject to dilution by adding 50 μL of water and were allowed to mixing by continuous vortex. The solution was centrifuged at 12,000 rpm for 15 min at 4 °C using a refrigerated centrifuge. The supernatant was collected for subsequent analysis. For method validation, quality control (QC) samples were prepared by pooling equal volumes of all individual urine samples to assess analytical consistency. LC-MS/MS analysis was performed on a Prominence LC-40 (Shimadzu Corporation, Kyoto, Japan) coupled to a Sciex ZenoTOF 7600 mass spectrometer (SCIEX, Framingham, MA, USA). A Kinetex C18 column (100 × 2.1 mm, 1.7 μm) maintained at 50 °C was employed with 2 μL injection volume. The mobile phases were 5 mM Ammonium formate in water (Phase A) and acetonitrile (Phase B) with 0.5 mL/min flow rate under the following gradient program: 1% B (0–1 min); 1–99% B (1–8 min); 99% B (8–10 min); 99–1% B (10–12 min). LC-MS/MS data were acquired in SCIEX OS software version 4.2 using a ZenoTOF 7600 system in data-dependent acquisition (DDA) mode. The key parameters of the ion source were set as follows: the spray voltage was 4.5 kV in the positive ion mode or −4.5 kV in the negative ion mode, Ion source gas 1 was 55 psi, Ion source gas 2 was 55 psi, Curtain gas was 35 psi, TOF start mass was 100 *m*/*z*, TOF stop mass was 1500 *m*/*z*, Source temperature was 500 °C. All samples were kept at 4 °C during analysis.

### 2.7. Microbiome Analysis

The collected mouse cecal contents were sent to 16S rRNA sequencing at Cosmos wisdom Co., Ltd. (Hangzhou, China). The primers 341-F (CCTACGGGNGGCWGCAG) and 806-R (GGACTACNVGGGTWTCTAAT) were selected to amplify the V3-V4 region of the 16S rRNA gene. Library construction and Illumina sequencing were completed by Cosmos wisdom Co., Ltd. according to the manufacturer’s instructions. For β-diversity analysis, NMDS based on Unweighted UniFrac distance was performed. PERMANOVA (Adonis, 999 permutations) was used to assess overall differences, and pairwise comparisons were conducted using the same method. For the Firmicutes/Bacteroidota (F/B) ratio, Kruskal–Wallis test followed by Dunn’s post hoc test was used for multiple-group comparisons, with statistical significance set at *p* < 0.05.

### 2.8. Potential Target of CT Extract Compounds

The composition of the prepared CT extract samples was determined by the LC-MS analysis. After being identified, the SMILES structures of the composition were obtained from the PubChem database [18]. Then the SMILES information was inserted into the SwissTargetPrediction platform (http://www.swisstargetprediction.ch/; accessed on 13 April 2025), HERB 2.0 Database (http://herb.ac.cn/; accessed on 17 April 2025), SEA platform (https://sea.bkslab.org/; accessed on 21 April 2025) to obtain their potential targets, and the target proteins with probability > 0 were selected. Combined with the target reported in the published research, the target proteins of CT extract were finally obtained.

#### 2.8.1. AKI-Related Target Screening

The AKI-related targets were obtained by database searching with “acute kidney injury” as the keyword. The online databases used in this step include GeneCards Data-base (https://www.genecards.org; accessed on 21 April 2025) [19] and DisGeNET Database (http://www.disgenet.org/) [20]. After removing duplicate information, the retained targets were imported into the Uniprot Protein Database (https://www.uniprot.org; accessed on 25 April 2025) [21] to obtain their corresponding gene name. To ensure transparency in target retrieval, the following stepwise filtering strategy was applied: (1) GeneCards targets were included based on a relevance score > 1.0, and DisGeNET targets were included based on a gene-disease association (GDA) score > 0.01, thresholds recommended for disease-specific target enrichment; (2) after deduplication and UniProt mapping, all retrieved targets constituted the disease-associated universe (11,789 entries); (3) intersection with CT extract compound targets (715 entries) yielded 667 high-confidence overlapping targets; and (4) hub targets were further prioritized by degree centrality (top 20 nodes) in the PPI network (String 12.0, confidence ≥ 0.4), identifying AKT1, TP53, MAPK1, and RELA as the most biologically central targets for downstream validation.

#### 2.8.2. Network Construction and Functional Enrichment Analysis

To study the interactions among targets, all overlapped target proteins of CT extract and AKI were uploaded to the string 12.0 database (https://cn.string-db.org/; accessed on 3 May 2025) to establish the PPI network. Species selection was “Homo sapiens”, and the association confidence was set to medium confidence (0.4). The PPI network was visualized with the help of Cytoscape 3.9.1 software [22]. Functional annotation and pathway enrichment were conducted using the DAVID Bioinformatics Resources (https://davidbioinformatics.nih.gov; accessed on 10 May 2025) (v2021 Update) to identify enriched Gene Ontology (GO) terms and KEGG pathways [23]. To elucidate the multi-target mechanism of CT extract, a bipartite Compound-Target-Pathway (C-T-P) network was constructed. This integrative approach allowed for the visualization of the systemic interactome, mapping the functional connectivity between bioactive phytochemicals, their primary protein targets, and the associated signaling cascades.

### 2.9. Molecular Docking Analysis

The CT extract compound and key target proteins selected from the C-T-P network analysis were used to perform molecular docking by BatchVinaGUI v2.2.0. Then, molecular docking was performed to investigate the potential interactions between bioactive compounds in CT extract and targets associated with AKI target. Literature searches were conducted to identify relevant targets, and docking was carried out to analyze the possible docking poses and binding affinities, providing insights into the binding activity between ligands and receptors. Based on previous identification of components in CT extract, the Structure-Data File (SDF) format files were downloaded from the Human Metabolome Database (HMDB) [24] and PubChem. Energy minimization was performed using Chem3D software (PerkinElmer, Waltham, MA, USA), and the optimized structures were subsequently converted into pdbqt format for molecular docking analysis.

Molecular docking was carried out with BatchVinaGUI v2.2.0. The X-ray crystal structures of RELA (PDB ID: 3RC0), AKT1 (PDB ID: 3O96), PI3KR1 (PDB ID: 3HHM), TP53 (PDB ID: 3ZME), MAPK14 (PDB ID: 2YIX), CDK2 (PDB IDs: 1B38), TNF (PDB ID: 2AZ5), GSK3B (PDB ID: 1Q5K), CASP3 (PDB ID: 3DEK), MAPK3 (PDB ID: 4QTB) were downloaded from the Protein Data Bank (https://www.rcsb.org; accessed on 13 July 2025). The workflow for BatchVinaGUI v2.2.0 software [25] is as follows. AutoDock Tools 1.5.7 was used for the preprocessing of both ligands and receptors. Subsequently, molecular docking was performed by the AutoDock Vina-GPU module [26]. The docking score affinity was used to screen bioactive phytochemicals and targets with better binding activity. Docking score Affinity < −5.0 kcal mol^−1^ indicates strong binding activity. Finally, the 3D structures of the binding modes were visualized in PyMOL 3.1.3 software. Note that molecular docking is an in-silico prediction and does not replace experimental validation of physical binding in vivo.

### 2.10. Biomarker Screening

Raw LC-MS data processing was conducted through Progenesis QI software v3.0 (Nonlinear Dynamics, Newcastle, UK) for peak picking, retention time alignment, and intensity normalization, producing finalized datasets with sample identifiers, peak tags, and intensity values. Unique features (retention time-*m*/*z* pairs) were aggregated by combining molecular adduct ions and isotope pattern deconvolution information to generate compound-specific “features” (retention time-*m*/*z* pairs), with summed intensities of corresponding ions. Normalization was applied universally using Progenesis QI. Metabolite annotation was performed within the software based on high-accuracy mass matches, isotopic pattern consistency, and manual validation of MS/MS spectral alignment (where feasible), with references from LipidMaps and HMDB. Pathway enrichment analysis was performed using MetaboAnalyst 5.0 [27], incorporating interquartile range (IQR) filtering, sum normalization, and Pareto scaling for data preprocessing. Multivariate pattern recognition, including principal component analysis (PCA) and orthogonal partial least squares discriminant analysis (OPLS-DA), were applied to identify group-specific metabolic differences. The significance values of the differential metabolites were confirmed with a triple screening criterion. Firstly, OPLS-DA variable importance in projection (VIP) scores > 1.0 was used to score candidates, then Student’s *t*-test *p*-values < 0.05 was applied to intergroup peak area comparisons, and finally, the biological significance was ensured with fold-change (FC) values ≥ 1.2 or ≤0.83 in intergroup intensity ratios. All differential metabolites reported in this study were putatively identified based on accurate mass and MS/MS spectral matching with public databases; authentic standards were not used for confirmation.

### 2.11. RNA Extraction and Real-Time Quantitative PCR

The extraction of total RNA content from kidney tissue was carried out with Trizol reagent kit (Tiangen, Beijing, China) according to the instructions from the manufacturer. Then, reverse transcription for genomic DNA clearance was performed using approximately 500 ng RNA with the help of FastQuant RT Kit (with gDNase, Tiangen, China). The reactions of real-time quantitative PCR were performed using the MonAmp™ ChemoHS qPCR Kit (Monad Biotech, Wuhan, China) to detect mRNA expression levels of relevant genes. Finally, relative expression level was measured by employing the 2^−ΔΔCt^ reference formula, with GAPDH used as a house keeping gene.

### 2.12. Statistical Analysis

Data analyses were mainly conducted on GraphPad Prism 7.0 (GraphPad Software Inc., San Diego, CA, USA), and the data was presented as mean ± SEM. The intergroup comparisons were carried out with one-way ANOVA, followed by post hoc test (Tukey’s test). Statistical significance was presented with *p* < 0.05, with thresholds denoted as * *p* < 0.05, ** *p* < 0.01 and *** *p* < 0.001.

## 3. Results

### 3.1. Phytochemical Profiling of CT Extract

The characterization of the bioactive matrix of the CT extract was carried out through quantitative phytochemical analysis. The total polysaccharides, analyzed using the phenol sulfuric acid method, were found to be 62.89%. Moreover, 13 major phytoconstituents (Table S1) were quantified, and Hydroxysafflor Yellow A was found to be the major quinochalcone at 3046.4 μg/g. On the other hand, a wide range of flavonoids was detected in the extract, such as Rutin (184.49 μg/g), Astragalin (161.76 μg/g), Isoquercitrin (81.52 μg/g), and Kaempferol-3-O-rutinoside (71.31 μg/g). The results of this biologically active matrix of the CT extract suggested the basis for exploring its role in systemic renoprotection.

### 3.2. CT Extract Improves Renal Injury in Mice

**Figure 1:**
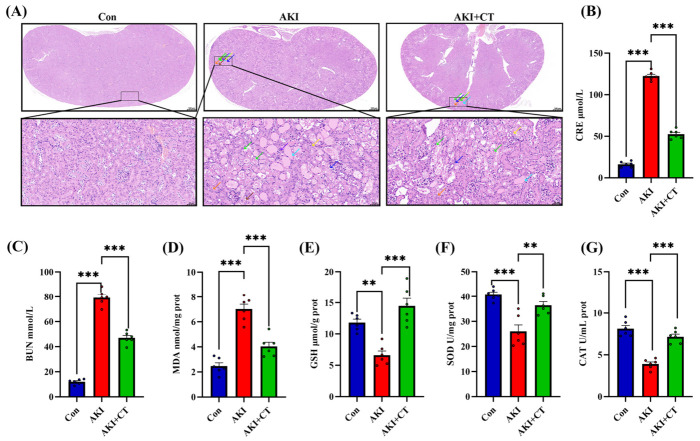
Safflower (CT) extract ameliorates cisplatin-induced renal injury and oxidative stress. (**A**) Representative images of renal histopathology (H&E and PAS staining). (**B**,**C**) Serum levels of biochemical markers: (**B**) creatinine (CRE) and (**C**) Blood Urea Nitrogen (BUN). (**D**–**G**) Assessment of renal redox homeostasis: (**D**) Malondialdehyde (MDA) content, (**E**) Glutathione (GSH) levels, (**F**) Superoxide Dismutase (SOD) activity, and (**G**) Catalase (CAT) activity. Data are presented as mean ± SEM (n = 6); see Methods for sample allocation. Significance was determined via one-way ANOVA followed by Tukey’s post hoc test; ** *p* < 0.01, *** *p* < 0.001 vs. the cisplatin-treated group.

Based on the high levels of the main components of CT extract (flavonoids and HSYA, known to possess antioxidants and anti-inflammatory activities), we further tested their therapeutic potential in vivo. To assess the potential role of CT extract in AKI caused by CDDP, we used an AKI animal model. The histopathology of kidney tissues indicated normal kidney structure in normal mice while the AKI group showed swollen tubular epithelial cells with vacuolar degeneration and severe necrosis, along with the presence of protein casts. CT extract administration resulted in a marked improvement in renal tissue architecture, including a pronounced reduction in tubular epithelial necrosis and protein cast formation; however, the kidney structure did not fully return to that of normal controls (Figure 1A). Regarding the quantification of kidney damage scores, we performed a semiquantitative histopathological scoring of H&E-stained sections using a validated scoring system (score 0–4) that evaluates tubular necrosis, cast formation, and loss of brush border. The mean damage scores were: Control: 0.17 ± 0.17; AKI: 3.50 ± 0.22; and AKI+CT: 1.83 ± 0.31 (mean ± SEM, n = 6). Biochemical analysis of serum markers revealed that cisplatin-induced elevations in CRE and BUN were significantly ameliorated following CT extract administration (Figure 1B,C). This reduction in functional biomarkers demonstrates the potent renoprotective effect of the safflower extract in the acute phase of cisplatin-induced injury.

Additionally, we examined changes in oxidative stress-related markers in renal tissue. Results showed that compared with the Control group, MDA levels were significantly increased, while GSH, SOD, and CAT levels were significantly reduced in the kidneys of AKI mice. Following CT extract treatment, MDA levels decreased significantly, while GSH, SOD, and CAT levels increased significantly, indicating that CT extract may improve renal injury in AKI mice by alleviating oxidative stress (Figure 1D–G).

### 3.3. CT Extract Reconfigures the Systemic Metabolic Landscape in AKI Mice

**Figure 2:**
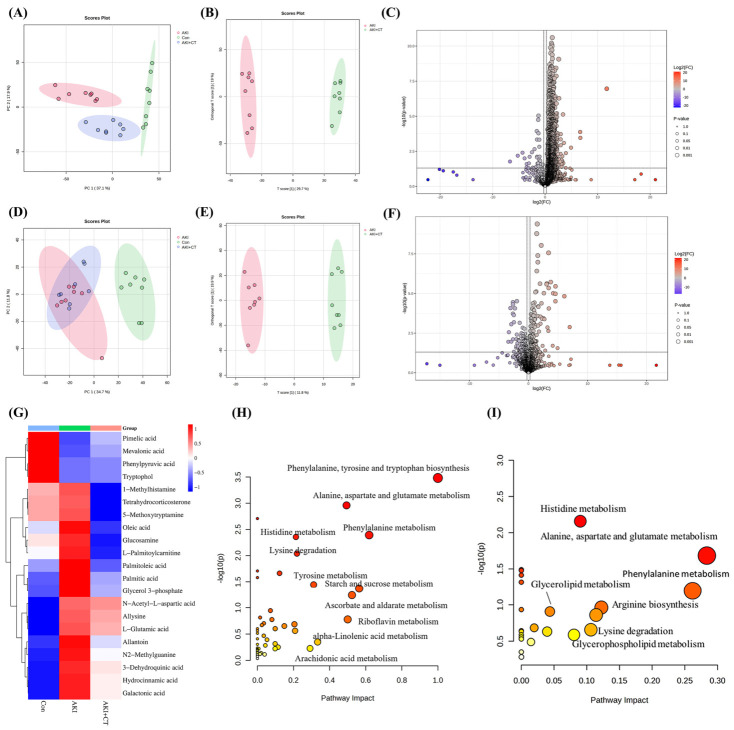
Urinary metabolomics profiling uncovers metabolic reprogramming by CT extract. (**A**–**C**) Multivariate and univariate analysis for positive-ion mode: (**A**) Scores plot obtained via PCA indicating metabolic segregation of samples globally; (**B**) Scores plot obtained via OPLS-DA indicating metabolic clustering of the sample groups distinctly; (**C**) Volcano plot revealing differentially regulated metabolites in terms of criteria of *p*-value (<0.05) and log2 fold change (|log2 FC| > 0.26). (**D**–**F**) Multivariate and univariate analysis for negative-ion mode: (**D**) Scores plot obtained via PCA depicting metabolic shift to Control group (Var PC1 = 34.7%); (**E**) Scores plot obtained via OPLS-DA validating sample groups segregation; (**F**) Volcano plot depicting differential metabolic characteristics. (**G**) Hierarchical clustering heatmap depicting 21 high-confidence differential metabolites and restoration effects of CT extract on AKI-specific metabolic signature. (**H**,**I**) KEGG pathway enrichment analysis of differential metabolites. Bubble plots show pathway enrichment for (**H**) AKI vs. Control and (**I**) AKI+CT vs. Control comparisons, generated using MetaboAnalyst. The *x*-axis represents pathway impact (derived from pathway topology analysis), and the *y*-axis represents statistical significance (−log10(*p*)). Bubble size is proportional to pathway impact, and color intensity reflects the −log10(*p*) value, with darker red indicating greater statistical significance.

To evaluate the systemic metabolic impact of CT extract, we performed untargeted LC-MS profiling of urinary metabolites (Figure S1). Multivariate analysis in both positive and negative-ion modes revealed a profound divergence between the Control and AKI groups, with the latter showing a distinct spatial segregation along PC1 (34.7% variance; Figure 2A,D). Importantly, the application of CT extract induced a significant metabolic shift away from the AKI cluster; however, the CT group did not fully merge with the Control cluster, instead forming an intermediate cluster between AKI and Control (Figure 2A,D). The CT group forms an independent cluster representing a “repair-specific intermediate state” between AKI and Control, rather than a complete return to normality. The inter-group distance between CT and Control is substantially smaller than between AKI and Control, confirming a positive shift toward the Control metabolic phenotype. This partial restoration trend was also supported by OPLS-DA parameters (Figure 2B,E). Filtering using volcano plots at *p* < 0.05 and FC > 1.2 indicated a significant change in the metabolic profile, where 2940 and 1919 metabolites were differently regulated in the positive and negative modes, respectively (Figure 2C,F). Heatmap analysis of discriminatory metabolites showed that the AKI profile, with high levels of lipid metabolites (L-palmitoylcarnitine, oleic acid, and palmitoleic acid) as well as stress metabolites (allantoin and 5-methoxytryptamine), was reversed by CT extract treatment (Figure 2G). On the other hand, AKI-induced reduction in the level of organic acids such as pimelic acid and mevalonic acid was partially increased by CT extract treatment, although they did not fully return to Control levels; nevertheless, the CT-treated samples shifted closer to the control branch.

Metabolic pathway enrichment analysis (KEGG) provided an additional description of the effects of this restoration. AKI featured disturbances in metabolic pathways associated with amino acid synthesis (tyrosine, phenylalanine, and tryptophan) and energy metabolism (Figure 2H, Table S2, putatively identified, see Section 2). The application of CT extract was able to successfully re-regulate these processes, with the strongest recovery of metabolism seen in histidine metabolism, alanine-aspartate-glutamate metabolism, and phenylalanine metabolism (Figure 2I, Table S3, putatively identified, see Section 2). Overall, these results show that CT extract is able to reverse disturbances in metabolism induced by AKI.

### 3.4. Effects of CT Extract on Gut Microbiota in AKI Mice

**Figure 3:**
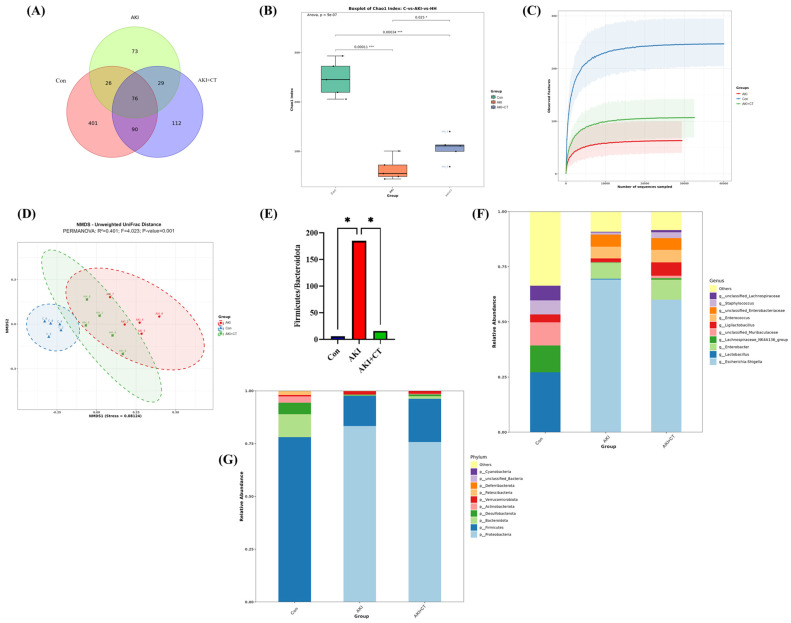
CT extract ameliorates cisplatin-induced renal injury by restoring gut microbial ecology and functional homeostasis. (**A**) Venn diagrams of gut microbiota in Control, AKI, and AKI+CT groups. (**B**) α diversity. (**C**) Dilution curve. (**D**) NMDS analysis. PERMANOVA: overall *p* = 0.001; pairwise comparisons: CT vs. AKI *p* = 0.038, CT vs. Control *p* = 0.013, AKI vs. Control *p* = 0.007. (**E**) Changes in Firmicutes/Bacteroidetes (F/B) ratio. (**F**) Microbial composition at genus level. (**G**) Microbial composition at phylum level. Data are presented as mean ± SD. * *p* < 0.05, *** *p* < 0.001. LEfSe analysis, and KEGG enrichment results are provided in Supplementary Figures S3–S5.

Given the established role of the gut–kidney axis in AKI and our metabolomic findings involving microbial-modulated pathways (e.g., bile acid and phenylalanine metabolism), we investigated whether CT extract renoprotection was associated with modulation of the intestinal microenvironment. The analysis of cecal 16S rRNA sequencing revealed distinct microbial signatures across groups. For instance, the distribution of gut microbiota across the Control, AKI, and CT extract groups revealed 76 operational taxonomic units (OTUs). While cisplatin (CDDP) treatment induced a profound collapse in the gut microbial α-diversity, evidenced by a significant reduction in the Chao1 index and unique OTU counts, CT extract intervention partially attenuated this loss of diversity. Similarly, the dilution curve analysis revealed markedly lower microbial abundance in the AKI group compared to the Control group, with modest recovery following CT extract treatment, suggesting CT extract moderately mitigates AKI-induced reduction in microbial abundance (Figure 3A–C). Non-metric multidimensional scaling (NMDS) further demonstrated that while AKI samples exhibited high dispersion (indicating dysbiosis), CT extract-treated profiles showed a directional shift toward the Control group, suggesting a partial structural modulation of dysbiosis (Figure 3D). PERMANOVA based on Unweighted UniFrac distance confirmed significant differences among the three groups (*p* = 0.001). Notably, the pairwise comparison CT vs. AKI showed a statistically significant difference (*p* = 0.038), but the effect size (R^2^ = 0.196) was relatively modest, indicating a distinguishable yet limited magnitude of community shift. This is consistent with the NMDS plot where CT samples partially overlap or cluster near the AKI group, rather than being completely separated. In contrast, Control vs. AKI (R^2^ = 0.438, *p* = 0.007) and CT vs. Control (R^2^ = 0.36, *p* = 0.013) showed larger effect sizes, indicating more pronounced community shifts. Collectively, these results suggest that CT treatment induces a significant but incomplete alteration of the gut microbiota, driving a partial shift away from the AKI-disturbed state without fully restoring the community to the Control profile. These statistical results are shown in the legend of Figure S2.

Furthermore, phylum-level composition revealed *Firmicutes* and *Bacteroidetes* as dominant phyla in the Control group. Conversely, significant spike of proteobacteria was observed while Firmicutes were found to be decreased. After the CT extract application, proteobacteria levels decreased while Firmicutes rebounded, suggesting CT extract alleviates phylum-level dysbiosis induced by AKI (Figure 3G). The Firmicutes to Bacteroidetes (F/B) ratio was used to denote gut microbiota dysbiosis. Likewise, the F/B ratio was found to be significantly high in the AKI group compared to the Control group, which reduced after CT extract administration, restoring microbial balance in the gut (Figure 3E). Further investigations at the genus level and via LEfSe analysis provided deeper insights. Pro-inflammatory pathogens like *Escherichia-Shigella*, *Enterococcus*, and *Staphylococcus*, along with their families *Enterobacteriaceae* and *Morganellaceae*, were dominant in the AKI group (Figure 3F, Figures S3 and S4). In contrast, CT extract suppressed these opportunistic pathogens while promoting the growth of beneficial taxa, specifically unclassified_*Muribaculaceae* and *Ligilactobacillus*.

To integrate taxonomic diversity towards metabolic changes, we employed PICRUSt2 functional prediction. Compared to the AKI group, CT extract treatment significantly upregulated pathways involved in primary and secondary bile acid biosynthesis, flavonoid metabolism, and streptomycin biosynthesis (Figure S5). These results suggest that the renoprotective effects of CT extract are associated with a functional reprogramming of the gut microbiota, particularly in pathways that intersect with host redox and metabolic signaling.

### 3.5. Network Pharmacology Analysis of CT Extract-AKI

**Figure 4:**
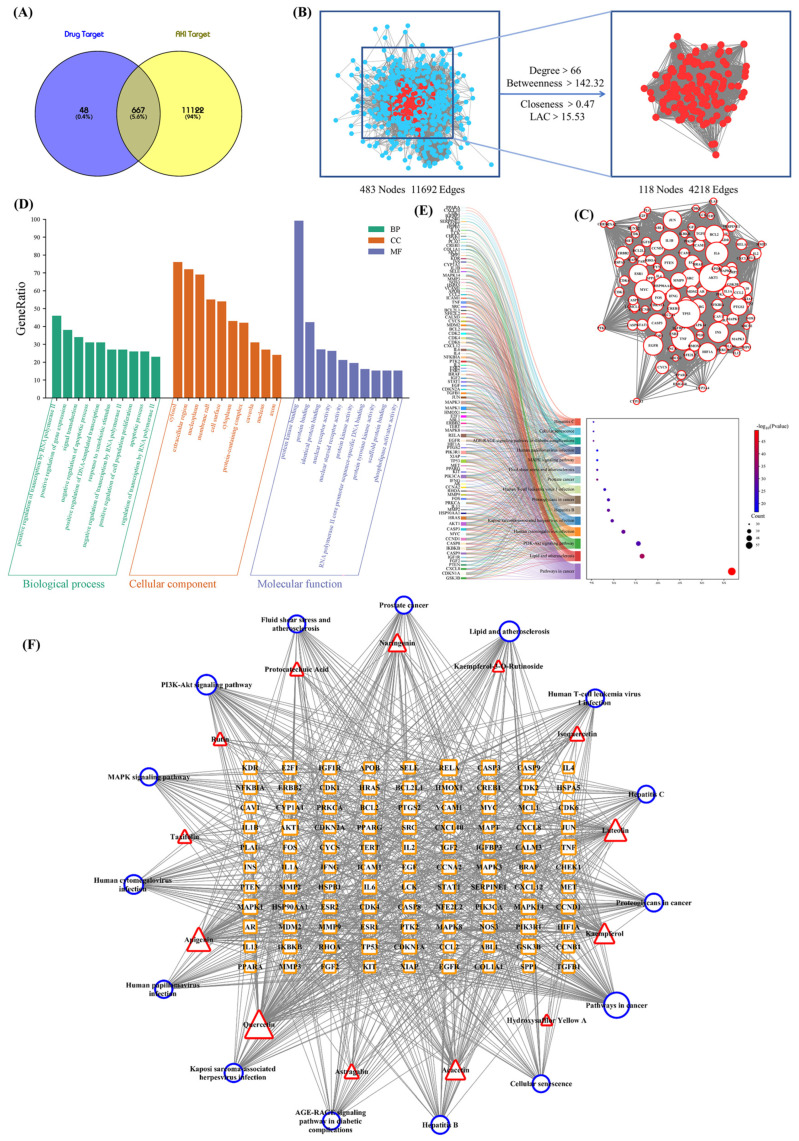
Network pharmacology analysis of CT extract for treating kidney injury. (**A**) Venn diagram of the intersection between components and disease targets. (**B**) Screening method for intersecting targets. (**C**) Protein interaction network. (**D**) Gene Ontology enrichment analysis. (**E**) KEGG pathway enrichment analysis. (**F**) Component-pathway-target network diagram.

To decipher the direct molecular targets of the bioactive components in CT extract that might underlie the observed phenotypic, metabolic, and microbial improvements, we employed an integrative network pharmacology approach. Using the chemical constituents of CT extract collected from LC-MS data, we screened databases and identified up to 715 relevant targets. After removing duplicate targets, we identified 11,789 targets associated with kidney injury (retrieved from GeneCards with relevance score > 1.0 and DisGeNET with GDA score > 0.01; these broad thresholds were intentionally inclusive to avoid missing biologically relevant targets, with subsequent intersection and PPI topology analysis serving as the primary filtering steps). Subsequently, we cross-referenced drug targets with disease targets using an online Venn diagram, revealing 667 overlapping targets (Figure 4A). Using Cytoscape 3.9.1, we constructed a component-target network, performed PPI analysis on key targets, and visualized a Component–Target–Pathway (CTP) diagram. AKT1, TP53, MAPK, and EGFR emerged as critical participants (Figure 4B,C).

GO functional annotation revealed key genes predominantly enriched in Biological Process (BP) categories such as positive regulation of transcription by RNA polymerase and negative regulation of apoptotic process; Cellular Component (CC) categories including cytosol and extracellular region; and Molecular Function (MF) categories like protein kinase binding and nuclear receptor activity (Figure 4D). KEGG enrichment analysis also revealed that key genes were predominantly enriched in the PI3K-AKT signaling pathway and MAPK signaling pathway, suggesting that the therapeutic potential of CT extract may be associated with these pathways (Figure 4E). The “Component–Target–Pathway (C–T–P)” diagram was generated from LC–MS analysis and network pharmacology. Core compounds in CT extract, including Rutin, Kaempferol-3-O-rutinoside, Quercetin, Hydroxysafflor yellow A, and Kaempferol, target key AKI-related proteins such as RELA, AKT1, and MAPK1. Through these interactions, CT extract exerts synergistic anti-inflammatory, antioxidant, and anti-apoptotic effects. The effects are associated with the PI3K–AKT, MAPK, and AGE–RAGE signaling pathways. This systematically elucidates the multi-component, multi-target, and multi-pathway renal protective mechanism of CT extract (Figure 4F, Tables S4 and S5).

### 3.6. Predicted Binding Interactions Between Core Bioactive Compounds and Therapeutic Targets

**Figure 5:**
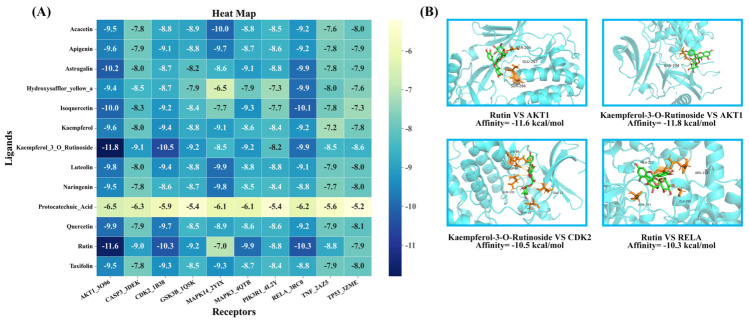
Molecular docking-based validation of binding affinities between CT extract bioactive substances and core signaling targets. (**A**) Binding affinity heatmap illustrating the interaction landscape between 13 signature compounds and the top-ranked protein targets. Color intensity reflects the binding energy (kcal/mol); lower binding energies indicate greater thermodynamic stability and stronger binding affinity. (**B**) Illustrations of 3D docking complexes show the interactions between key bioactive compounds and their targets: Rutin with AKT1 (binding energy: −11.6 kcal/mol), Kaempferol-3-O-rutinoside with AKT1, Kaempferol-3-O-rutinoside with CDK2, and Rutin with RELA (p65). Hydrogen bonds, hydrophobic interactions, and the specific amino acids within the binding pockets are highlighted to demonstrate structural complementarity.

To further validate the potential interactions between the core bioactive compounds identified in CT extract and the key targets predicted by network pharmacology, we performed molecular docking analysis. The results indicate that compounds such as Rutin, Kaempferol-3-O-Rutinoside, and Quercetin interact with core target genes AKT1_3O96, CASP3_3DEK, CDK2_1B38, GSK3B_1Q5K, MAPK14_2YIX, MAPK3_4QTB, PIK3R1_4L2Y, RELA_3RC0, TNF_2AZ5, and TP53_3ZME (Figure 5A, Table S6). Among these, Rutin and Kaempferol-3-O-Rutinoside exhibited the most significant binding efficiency toward disease targets. The binding energy between Rutin and AKT1 was −11.6 kcal/mol, with binding sites at SER-205, GLU-267, and SER-266. Kaempferol-3-O-rutinoside bound to AKT1 with an energy of −11.8 kcal/mol, with binding sites at ASN-204. Kaempferol-3-O-rutinoside bound to CDK2 with an energy of −10.5 kcal/mol with binding sites at LYS-89, ASP-86, GLN-131, THR-14, and GLU-12. The binding energy between Rutin and RELA is −10.3 kcal/mol, with binding sites at ALA-222, ARG-152, ASN-251, GLY-298. This indicates that CT extract components may exert significant effects by binding to these targets (Figure 5B).

### 3.7. CT Extract Alleviates Kidney Injury by Modulating the PI3K/Akt Pathway and Antioxidant-Related Genes

**Figure 6:**
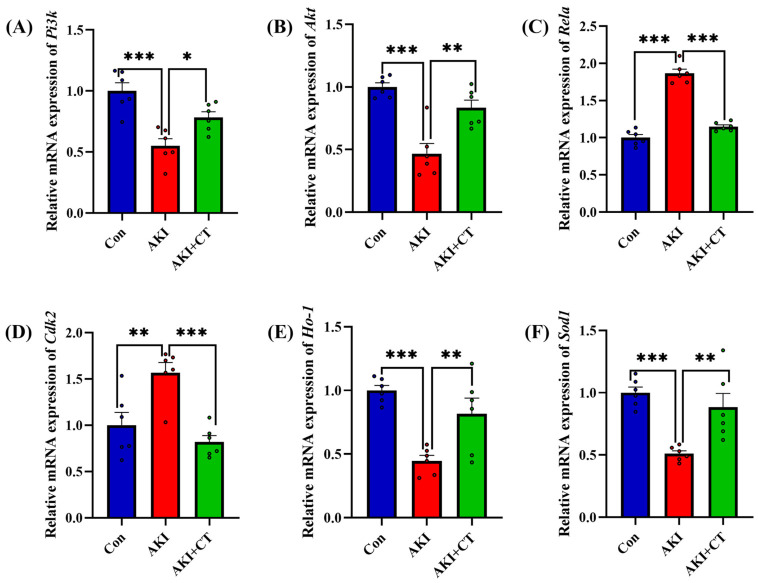
CT extract modulates the PI3K/Akt signaling axis and enhances the renal antioxidant transcriptional program. (**A**–**F**) Relative mRNA expression levels of core regulatory and effector genes in murine renal tissue: (**A**) *Pi3k*, (**B**) *Akt*, (**C**) *Rela*, (**D**) *Cdk2*, (**E**) *Ho-1*, and (**F**) *Sod1*. Data are presented as mean ± SEM (n = 6 per group); see Methods for sample allocation. Statistical significance: * *p* < 0.05, ** *p* < 0.01, *** *p* < 0.001 vs. the AKI group.

Based on the strong binding affinities predicted by molecular docking, particularly for targets within the PI3K-AKT and NF-κB pathways (Figure 5), we experimentally validated the expression of key regulatory genes in renal tissue. RT-qPCR analysis revealed that cisplatin-induced AKI triggered a profound transcriptional suppression of the PI3K/Akt pathway, with significant downregulation of *Pi3k* and *Akt* mRNA levels compared to the Control group (Figure 6). This was accompanied by a marked decrease in the expression of downstream antioxidant effectors, including *Heme Oxygenase-1* (*Ho-1*) and *Superoxide Dismutase 1* (*Sod1*).

Conversely, CT extract intervention significantly rescued the expression of *Pi3k*, *Akt*, *Ho-1*, and *Sod1*, suggesting a strong transcriptional upregulation of the Nrf2-mediated antioxidant program. Furthermore, CT extract effectively attenuated the AKI-associated upregulation of the pro-inflammatory mediator *RelA* (*p65*) and the cell-cycle regulator *Cdk2*, both of which are critical drivers of renal pathological progression. The upregulation of Ho-1 and Sod1 is consistent with the induction of the Nrf2-mediated antioxidant program, a key downstream effect of PI3K/AKT signaling at the transcriptional level. These results suggest that the renoprotective efficacy of CT extract is associated with the coordinated modulation of the PI3K/Akt/Nrf2 axis, which suppresses inflammatory signaling while enhancing the cellular redox defense capacity.

## 4. Discussion

This study provides that the first integrated multi-omics elucidates the renoprotective mechanisms with a particular emphasis on systemic immunomodulation via the gut–kidney axis, combining urine untargeted metabolomics, 16S rRNA sequencing, network pharmacology, and molecular validation. Our results demonstrate that CT extract confers protection through a concerted “multi-component–multi-target–multi-pathway” strategy, which extends beyond direct cellular defense to reprogram systemic host–microbiota co-metabolism and modulate central pro-survival signaling. These findings provide robust scientific evidence supporting the discovery of CT extract as a chemo-protective adjuvant.

First, CT extract directly intercepts the core drivers of cisplatin nephrotoxicity, oxidative stress and metabolic paralysis. Cisplatin-induced ROS production triggers a malicious cycle of lipid peroxidation, inflammation, and tubular cell death [28,29]. Consistently, we found CT extract significantly alleviated histological damage and renal dysfunction (reduced CRE/BUN), while potently attenuating oxidative stress (decreased MDA, restored GSH/SOD/CAT). These results are found consistent with the previously established antioxidant properties of safflower and its bioactive components [30,31]. Significantly, our untargeted metabolomics analysis showed that CT extract altered the disturbances in metabolic homeostasis caused by cisplatin. This involved the normalization of 21 metabolites, including amino acids involved in key energy and biosynthesis pathways such as histidine, alanine-aspartate-glutamate, and phenylalanine. By restoring fatty acid β-oxidation and replenishing TCA cycle substrates, CT extract may help alleviate tubular energy failure, a process that likely works synergistically with its antioxidant activity to prevent cell death [32,33].

A novel and pivotal finding of this study is that CT extract remodels the gut–kidney axis, representing a systemic immunomodulatory mechanism. We observed that CT extract reversed cisplatin-induced dysbiosis by restoring α-diversity, reducing the Firmicutes/Bacteroidota ratio, suppressing pathobionts (*Escherichia-Shigella*, *Enterococcus*), and enriching beneficial taxa (*Ligilactobacillus*, *Muribaculaceae*). Notably, the degree of microbial reconfiguration correlated with host metabolic improvement, supporting a functional “microbiota–metabolism” interaction as a key therapeutic link. PICRUSt2 analysis suggested an upregulation of microbial pathways for flavonoid and secondary bile acid synthesis, suggesting at enhanced microbiota–host co-metabolism. This positions CT extract alongside other natural products that confer renal protection via modulating the gut–kidney axis and its associated inflammatory signaling [34,35]. The dramatic increase in the Firmicutes/Bacteroidota (F/B) ratio observed in the AKI group (nearly 200) and its significant reduction by CT extract raises the question of whether this effect is a direct prebiotic action or an indirect consequence of reduced systemic inflammation. We propose that both mechanisms likely contribute. On one hand, the abundant polysaccharides (62.89%) and flavonoids in CT extract may directly act as prebiotics, promoting beneficial bacteria and suppressing opportunistic pathogens. On the other hand, CT extract attenuates cisplatin-induced systemic inflammation (e.g., downregulation of renal Rela/NF-κB and reduced pro-inflammatory cytokines), which may indirectly improve the gut microenvironment by alleviating inflammatory damage and hypoxia, thereby facilitating microbial reconstitution. Thus, the modulation of the F/B ratio by CT extract probably involves synergistic contributions from both direct prebiotic and indirect anti-inflammatory effects. By repairing the gut ecosystem, CT extract may reduce the burden of gut-derived uremic toxins and pro-inflammatory signals (e.g., LPS) that exacerbate renal injury through pathways like TLR4/NF-κB.

The interaction of various active components at critical signaling nodes has been unraveled using network pharmacology. Analysis identified 667 common targets for both CT extract and AKI, with the PI3K-AKT and MAPK pathways playing key roles. Molecular docking suggested strong binding of major flavonoids, including Rutin and Kaempferol-3-O-rutinoside, to essential targets such as AKT1, CDK2, and RELA. These computational predictions were experimentally validated: RT-qPCR confirmed upregulation of Pi3k, Akt, Ho-1, and Sod1, alongside downregulation of Rela and Cdk2. The PI3K-AKT axis, when transcriptionally upregulated, can act as a master regulator, capable of suppressing apoptosis and promoting NRF2-driven antioxidant responses [36]. The effectiveness of CT extract is therefore likely associated with the combined action of its flavonoids, each known for their kidney-protective effects through related mechanisms [17,37,38].

It is important to note that the molecular docking results presented here (e.g., binding energies of −11.6 and −11.8 kcal/mol) are purely computational. They do not prove that rutin or kaempferol-3-O-rutinoside physically binds to AKT1, CDK2, or RELA in the mouse kidney. Orthogonal experimental methods, such as surface plasmon resonance (SPR), cellular thermal shift assays (CETSA), or site-directed mutagenesis coupled with biophysical binding assays, are required to confirm actual target engagement in vivo. Therefore, our docking data should be interpreted as hypothesis-generating rather than conclusive.

This study provides a strong multi-omics foundation for the translational development of CT extract. However, the mouse model used cannot fully replicate the complexity of cancer patients undergoing chemotherapy. While we observed consistent changes in the mRNA levels of several PI3K/Akt-related genes, these transcriptional findings do not directly demonstrate pathway activation at the protein level. Importantly, due to the limited amount of kidney tissue available, the samples were fully consumed during the measurement of renal antioxidant markers, qPCR analysis, and histological staining. No residual tissue remained to perform Western blotting or phosphoproteomics analyses to confirm the phosphorylation status of key components such as Akt. Therefore, our conclusions regarding PI3K/Akt activation are primarily based on network pharmacology predictions and qPCR validation, within a broader multi-omics context that demonstrates concerted renoprotection at the metabolic, microbial, and transcriptional levels. While these multi-omics approaches provide convergent evidence, they may not fully capture the dynamic post-translational regulation of this signaling cascade. Future studies with dedicated sample collection and preservation protocols will be needed to validate these findings at the phosphoprotein level and to further dissect the underlying signaling dynamics. The precise in vivo pharmacokinetics of the bioactive components and the causal link between specific microbial changes and nephroprotection remain to be clarified. Several additional limitations of this study merit acknowledgment. First, this study exclusively utilized male C57BL/6J mice; sex differences in cisplatin nephrotoxicity are well documented, and the findings may not be directly generalizable to female subjects. Future studies should include both sexes to comprehensively evaluate sex-specific renoprotective effects of CT extract. Second, the gut microbiota changes observed are correlational, and causal inference will require fecal microbiota transplantation (FMT) or germ-free model experiments. Third, as already discussed, the molecular docking results remain computational and require experimental target engagement validation. Fourth, the translational relevance of the 40 mg/kg dose relative to human-equivalent doses remains to be established through formal pharmaco-kinetic-pharmacodynamic studies.

## 5. Conclusions

In the present study, we provide the first evidence that *Carthamus tinctorius* L. extract (CT) offers robust protection against cisplatin-induced acute kidney injury (AKI) through a coordinated gut–metabolome–kidney pathway. Our analysis showed that CT extract (i) restores systemic metabolic homeostasis by normalizing amino acid and energy metabolism; (ii) modulates gut microbiota by reducing pro-inflammatory pathogens and enhancing commensal bacteria; and (iii) transcriptionally upregulates PI3K/Akt/Nrf2 signaling-related genes, which may contribute to strengthening renal antioxidant defenses. This represents a shift from conventional single-target therapy toward a holistic systems-level approach. While further validation in tumor-bearing models and pharmacokinetic studies is needed, these findings provide a strong scientific rationale for using CT extract as a protective adjuvant against chemotherapy-induced nephrotoxicity.

## Data Availability

The original data generated in this study are part of an ongoing research project and therefore cannot be made publicly available at this stage. However, the data will be made available by the corresponding author upon reasonable request. Once the broader experimental framework of the ongoing project has been completed, the data will be deposited in a public repository and made openly accessible.

## Supplementary Materials

The following supporting information can be downloaded at: https://www.mdpi.com/article/10.3390/antiox15070855/s1, Table S1: 13 Compounds Content in CT Extract; Table S2: The information of the annotated urine metabolites; Table S3: The annotated information of biomarkers with reversal trend after CT extract treatment; Table S4: Feature parameters of CT extract compound in the G-T-P network; Table S5: Characteristic parameters of target proteins in the G-T-P network (Top 10); Table S6: Molecular docking results of compounds with disease targets; Figure S1: Representative Base Peak Chromatogram of urine sample extracts; Figure S2. Pairwise NMDS analysis of gut microbiota based on Unweighted UniFrac distance. (A) AKI vs. Control; (B) CT extract treated (AKI+CT) vs. AKI; (C) CT extract treated vs. Control. PERMANOVA (Adonis, 999 permutations) *p* values are indicated on each plot. Stress values: (A) <0.001, (B) 0.079, (C) 0.049. These pairwise comparisons support the three group NMDS analysis presented in Figure 3D; Figure S3: LEfSe analysis revealing phylogenetic distribution of differential taxa among groups; Figure S4: LEfSe analysis identifying discriminatory OTUs/ASVs among groups; Figure S5: KEGG enrichment analysis of differentially abundant microbial functions between the AKI and AKI+CT groups.

## Abbreviations

AKI	Acute Kidney Injury
CDDP	Cisplatin
CT extract	*Carthamus tinctorius* L. extract
BUN	Blood Urea Nitrogen
CAT	Catalase
CRE	Creatinine
GSH	Glutathione
MDA	Malondialdehyde
SOD	Superoxide Dismutase
16S rRNA	16S Ribosomal RNA
DDA	Data-Dependent Acquisition
FC	Fold Change
H&E	Hematoxylin and Eosin
HPLC	High-Performance Liquid Chromatography
LC-MS	Liquid Chromatography-Mass Spectrometry
OPLS-DA	Orthogonal Partial Least Squares Discriminant Analysis
OTU	Operational Taxonomic Unit
PCA	Principal Component Analysis
PPI	Protein–Protein Interaction
RT-qPCR	Reverse Transcription Quantitative Polymerase Chain Reaction
VIP	Variable Importance in Projection
C-T-P	Component–Target–Pathway
GO	Gene Ontology
KEGG	Kyoto Encyclopedia of Genes and Genomes
AKT1	Protein Kinase B
CDK2	Cyclin-Dependent Kinase 2
HO-1	Heme Oxygenase-1
MAPK1	Mitogen-Activated Protein Kinase 1
PI3K	Phosphatidylinositol 3-Kinase
RELA	Transcription factor p65 (NF-κB subunit)
SOD1	Superoxide Dismutase 1
ANOVA	Analysis of Variance
SEM	Standard Error of the Mean
