# Supplementary material for: Safflower Extract Ameliorates Cisplatin-Induced Acute Kidney Injury by Regulating Microbiota-Metabolic-Redox Nexus and PI3K–Akt/Nrf2 Pathway
Source: Antioxidants (Basel). 2026 Jul 7;15(7):855. doi: 10.3390/antiox15070855 (PMC13403974; doi:10.3390/antiox15070855)
Supplement: Supplementary file 1 [file antioxidants-15-00855-s001.zip › antioxidants-4331131-supplementary/antioxidants-4331131-supplementary-6.25/supplementary files/Supplementary_Materials (1).pdf]

Supplementary Material

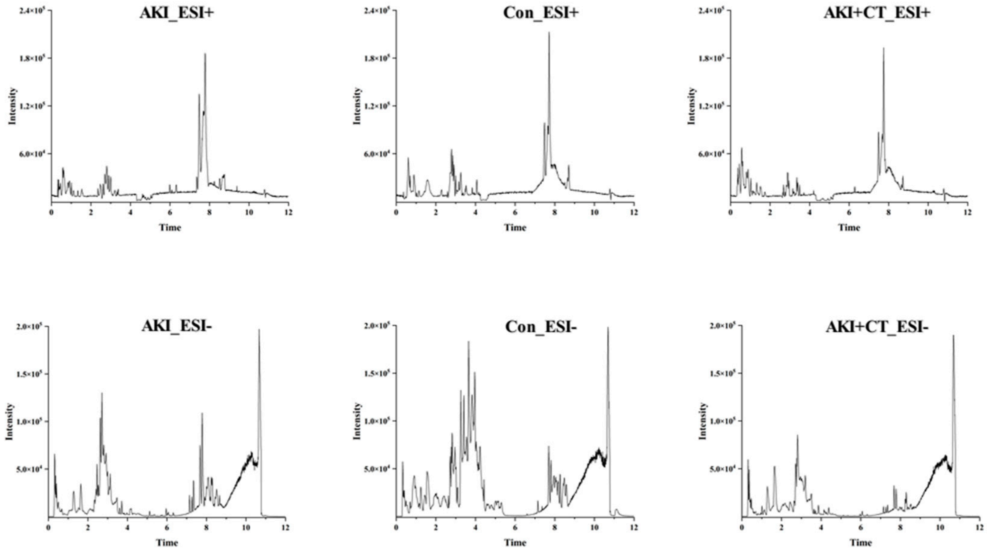

Figure S1. Representative Base Peak Chromatogram of urine sample extracts.

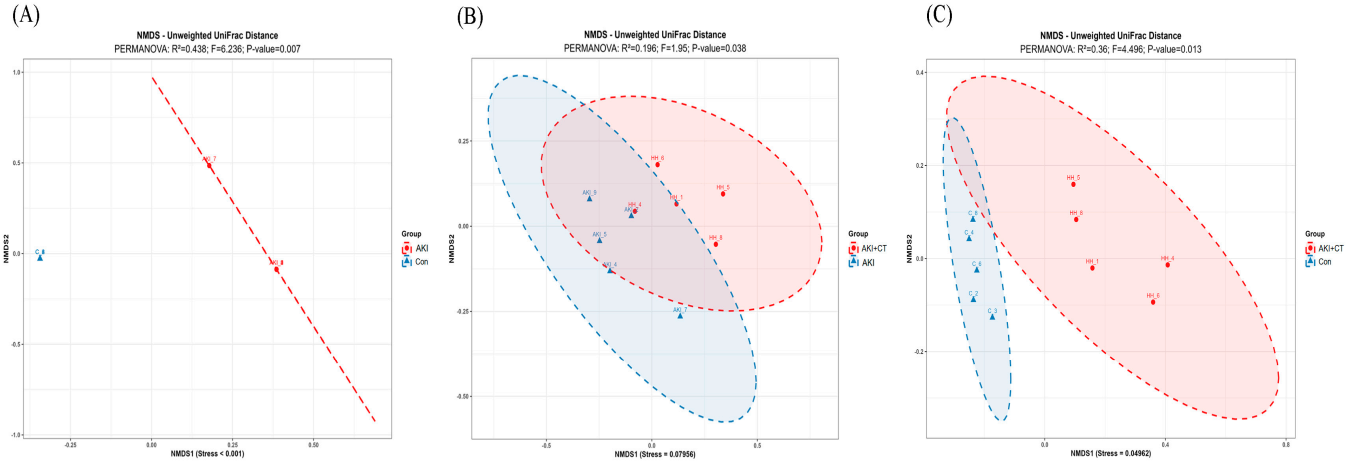

Figure S2. Pairwise NMDS analysis of gut microbiota based on Unweighted UniFrac distance. (A) AKI vs Control; (B) CT extract treated (AKI+CT) vs AKI; (C) CT extract treated vs Control. PERMANOVA (Adonis, 999 permutations) p values are indicated on each plot. Stress values: (A) <0.001, (B) 0.079, (C) 0.049. These pairwise comparisons support the three group NMDS analysis presented in Figure 3D.

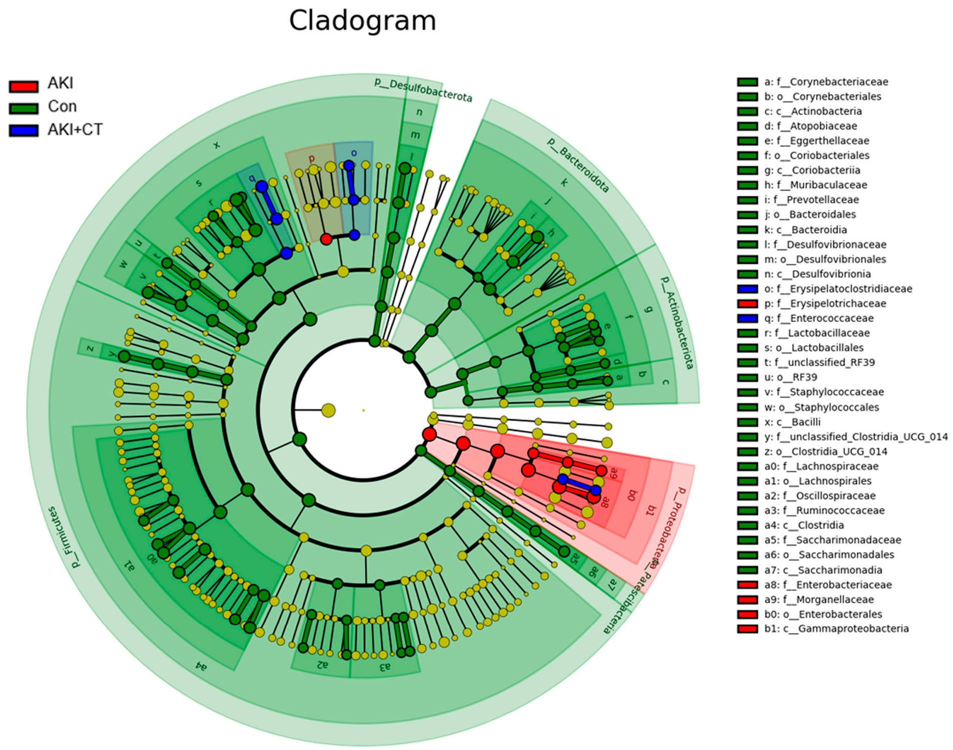

Figure S3. LEfSe analysis revealing phylogenetic distribution of differential taxa among groups.

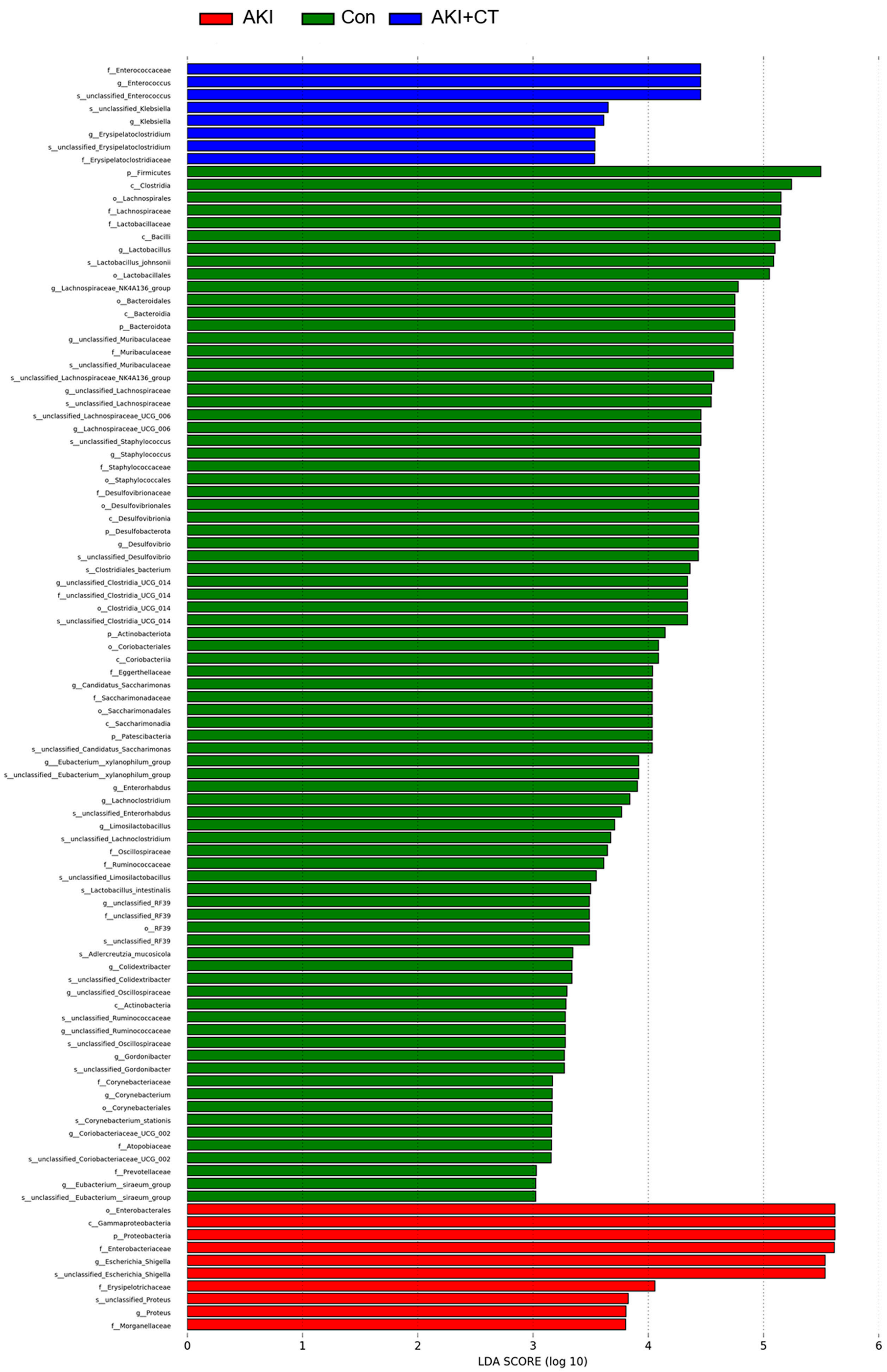

Figure S4. LEfSe analysis identifying discriminatory OTUs/ASVs among groups.

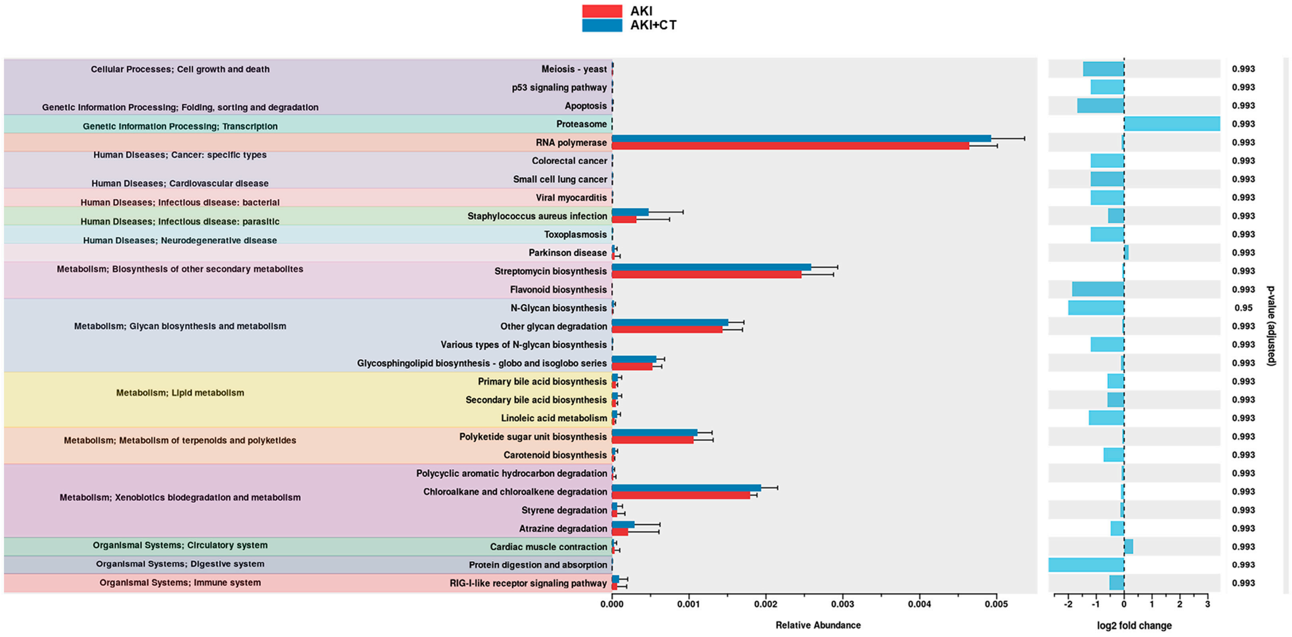

Figure S5. KEGG enrichment analysis of differentially abundant microbial functions between the AKI and AKI+CT groups.

Table S1： 13 Compounds Content in CT Extract

Compound	Content（μg/g）	Q1（Da）	Q3(Da）	EP（V）	CE（V）	CXP（V）
Hydroxysafflor yellow A	3046.4	611.15	491.1	-10	-30	-15
Rutin	184.49	609.14	301.03	-10	-30	-15
Astragalin	161.758	447.09	284.01	-10	-30	-15
Isoquercitrin	81.522	463.08	300.02	-10	-30	-15
Kaempferol-3-O-rutinoside	71.312	593.13	285.03	-10	-30	-15
Kaempferol	46.82	285.1	229.02	-10	-30	-15
Quercetin	26.062	301.06	150.97	-10	-30	-15
Protocatechuic acid	24.686	153.04	109.01	-10	-30	-15
Apigenin	21.652	269.14	117	-10	-30	-15
Luteolin	13.3926	285.08	133	-10	-30	-15
Naringenin	11.4734	271.12	119.02	-10	-30	-15
Taxifolin	0.468765	303.09	124.99	-10	-30	-15
Acacetin	0.00169665	283.12	268.02	-10	-30	-15

Table S2: The information of the annotated urine metabolites.

No.	Compound	Formula	HMDB	RT(min)	M/Z	Adducts	FC value	raw.pval	VIP value	Isotope Similarity
1	N-Acetyl-L-aspartic acid	$C_6H_9NO_5$	HMDB0000812	0.32	176.0661	[M+H]+	0.24	1.70E-06	1.44	98.08
2	Citric acid	$C_6H_8O_7$	HMDB0000094	0.33	230.9899	[M+K]+	4.51	6.35E-03	1.02	95.87
3	Galactonic acid	$C_6H_{12}O_7$	HMDB0000565	0.34	241.0289	[M+2Na-H]+	0.09	9.43E-04	1.17	96.14
4	Butyric acid	$C_4H_8O_2$	HMDB0000039	0.36	130.0857	[M+ACN+H]+	0.48	3.66E-04	1.23	97.19
5	N6,N6,N6-Trimethyl-L-lysine	$C_9H_{20}N_2O_2$	HMDB0001325	0.36	189.1595	[M+H]+	0.27	3.18E-10	1.53	99.21
6	Oxoglutaric acid	$C_5H_6O_5$	HMDB0000208	0.37	191.0199	[M+FA-H]-	2.51	2.24E-03	1.21	98.23
7	2-Furoic acid	$C_5H_4O_3$	HMDB0000617	0.37	111.0089	[M-H]-	2.65	1.63E-03	1.23	98.29
8	Creatine	$C_4H_9N_3O_2$	HMDB0000064	0.38	132.0765	[M+H]+	0.04	5.43E-05	1.32	98.54
9	Gluconolactone	$C_6H_{10}O_6$	HMDB0000150	0.38	177.04	[M-H]-	0.58	4.01E-05	1.4	96.07
10	Threonic acid	$C_4H_8O_5$	HMDB0000943	0.38	135.0297	[M-H]-	1.61	5.49E-04	1.29	95.39
11	D-Ribose	$C_5H_{10}O_5$	HMDB0000283	0.39	195.0511	[M+FA-H]-	0.44	1.21E-02	1.01	98.37
12	D-Fructose	$C_6H_{12}O_6$	HMDB0000660	0.41	215.0326	[M+Cl]-	0.12	9.45E-09	1.6	98.28
13	Biotin sulfone	$C_{10}H_{16}N_2O_5S$	HMDB0004818	0.41	321.0816	[M+FA-H]-	0.31	9.08E-06	1.47	96.21
14	2-Hydroxybutyric acid	$C_4H_8O_3$	HMDB0000008	0.42	103.0399	[M-H]-	0.08	2.17E-05	1.44	97.75
15	D-Glucuronic acid	$C_6H_{10}O_7$	HMDB0000127	0.42	175.0247	[M-H$_2$O-H]-	0.26	1.23E-04	1.39	98.3
16	3-Dehydroquinic acid	$C_7H_{10}O_6$	HMDB0012710	0.42	189.0403	[M-H]-	0.43	3.37E-07	1.54	97.99
17	D-Glucose	$C_6H_{12}O_6$	HMDB0000122	0.45	203.0523	[M+Na]+	0.02	1.60E-06	1.41	97.44
18	Glucose 6-phosphate	$C_6H_{13}O_9P$	HMDB0001401	0.48	298.9922	[M+K]+	4.74	3.13E-03	1.1	95.77
19	Allantoin	$C_4H_6N_4O_3$	HMDB0000462	0.49	203.0153	[M+2Na-H]+	0.25	1.95E-03	1.11	95.43
20	Isovaleric acid	$C_5H_{10}O_2$	HMDB0000718	0.54	144.1014	[M+ACN+H]+	4.42	2.20E-04	1.24	97.39
21	L-Acetylcarnitine	$C_9H_{17}NO_4$	HMDB0000201	0.58	204.1235	[M+H]+	0.04	1.82E-11	1.54	98.34
22	3-Methoxy-4-hydroxyphenylglycol glucuronide	$C_{15}H_{20}O_{10}$	HMDB0000496	0.59	378.1379	[M+NH$_4$]+	0.23	8.73E-04	1.19	96.18

23	Aminoadipic acid	$C_6H_{11}NO_4$	HMDB0000510	0.6	142.0509	[M-H2O-H]-	0.38	1.62E-03	1.22	98.12
24	Uridine	$C_9H_{12}N_2O_6$	HMDB0000296	0.63	267.0571	[M+Na]+	0.43	7.65E-05	1.32	96.59
25	L-Glutamine	$C_5H_{10}N_2O_3$	HMDB0000641	0.64	181.0377	[M+Cl]-	0.4	1.65E-04	1.38	97.11
26	1-Methylhistidine	$C_7H_{11}N_3O_2$	HMDB0000001	0.66	170.0919	[M+H]+	0.37	1.42E-05	1.37	96.28
27	Pimelic acid	$C_7H_{12}O_4$	HMDB0000857	0.69	319.1392	[2M-H]-	11.04	1.24E-06	1.53	95.31
28	Urocanic acid	$C_6H_6N_2O_2$	HMDB0000301	0.7	139.0497	[M+H]+	2.51	1.47E-03	1.12	97.98
29	N1-Acetylspermidine	$C_9H_{21}N_3O$	HMDB0001276	0.75	188.1754	[M+H]+	0.25	2.01E-03	1.12	97.13
30	Hypoxanthine	$C_5H_4N_4O$	HMDB0000157	0.77	181.0379	[M+FA-H]-	0.43	6.01E-06	1.49	95.75
31	Pantothenic acid	$C_9H_{17}NO_5$	HMDB0000210	0.77	218.1041	[M-H]-	2.09	2.64E-04	1.35	98.68
32	Pipecolic acid	$C_6H_{11}NO_2$	HMDB0000070	0.79	174.0772	[M+FA-H]-	3.12	4.90E-05	1.42	96.18
33	Dodecanedioic acid	$C_{12}H_{22}O_4$	HMDB0000623	0.79	265.1189	[M+Cl]-	10.96	1.56E-03	1.23	96.38
34	Allysine	$C_6H_{11}NO_3$	HMDB0001263	0.79	184.0278	[M+K]+	0.33	5.29E-03	1.04	98.82
35	6-Methylthioguanosine monophosphate	$C_{11}H_{16}N_5O_7PS$	HMDB0060413	0.79	374.0255	[M-H2O-H]-	0.14	2.96E-04	1.32	98.66
36	3,4-Dihydroxyphenylglycol	$C_8H_{10}O_4$	HMDB0000318	0.81	188.092	[M+NH4]+	0.35	2.03E-03	1.13	96.79
37	Glycolic acid	$C_2H_4O_3$	HMDB0000115	0.87	153.0406	[2M+H]+	6.41	1.38E-04	1.29	98.82
38	Epinephrine	$C_9H_{13}NO_3$	HMDB0000068	0.88	228.0876	[M+FA-H]-	0.44	3.58E-03	1.17	95.49
39	5-Hydroxylysine	$C_6H_{14}N_2O_3$	HMDB0000450	0.89	204.1338	[M+ACN+H]+	0.09	4.06E-09	1.52	99.38
40	Porphobilinogen	$C_{10}H_{14}N_2O_4$	HMDB0000245	0.95	227.1019	[M+H]+	0.4	6.06E-05	1.32	97.55
41	Pyrocatechol	$C_6H_6O_2$	HMDB0000957	1	109.0294	[M-H]-	4.5	1.85E-03	1.22	98.67
42	Hippuric acid	$C_9H_9NO_3$	HMDB0000714	1.1	162.0546	[M+H-H2O]+	7.42	5.81E-05	1.34	95.12
43	L-Tyrosine	$C_9H_{11}NO_3$	HMDB0000158	1.13	162.0558	[M-H2O-H]-	16.27	1.97E-05	1.44	97.91
44	Adenine	$C_5H_5N_5$	HMDB0000034	1.17	116.0352	[M-H2O-H]-	0.25	5.14E-04	1.28	95.58
45	L-Phenylalanine	$C_9H_{11}NO_2$	HMDB0000159	1.22	164.0707	[M-H]-	0.56	6.89E-04	1.25	95.97
46	5-Hydroxyindoleacetic acid	$C_{10}H_9NO_3$	HMDB0000763	1.23	236.0596	[M+FA-H]-	0.1	3.79E-04	1.3	96.94
47	Mevalonic acid	$C_6H_{12}O_4$	HMDB0000227	1.24	129.0555	[M-H2O-H]-	2.52	5.11E-06	1.5	96
48	N2-Methylguanine	$C_6H_7N_5O$	HMDB0006040	1.33	166.0732	[M+H]+	0.42	3.32E-04	1.25	96.8

49	Hydroxyphenylacetylglycine	$C_{10}H_{11}NO_4$	HMDB0000735	1.46	208.0645	[M-H]-	0.22	1.98E-05	1.44	96.33
50	Isovaleraldehyde	$C_5H_{10}O$	HMDB0006478	1.46	131.0712	[M+FA-H]-	0.18	6.74E-04	1.27	96.77
51	Dopamine	$C_8H_{11}NO_2$	HMDB0000073	1.49	134.061	[M-H2O-H]-	6.75	8.75E-03	1.08	98.72
52	N1-Methyl-2-pyridone-5-carboxamide	$C_7H_8N_2O_2$	HMDB0004193	1.52	175.0473	[M+Na]+	0.46	9.19E-05	1.29	97.07
53	3-Hydroxyanthranilic acid	$C_7H_7NO_3$	HMDB0001476	1.53	136.0389	[M+H-H2O]+	0.44	5.29E-05	1.34	98.57
54	Xanthurenic acid	$C_{10}H_7NO_4$	HMDB0000881	1.54	204.03	[M-H]-	2.89	1.19E-03	1.23	98.08
55	Benzoic acid	$C_7H_6O_2$	HMDB0001870	1.55	105.0332	[M+H-H2O]+	2.66	2.29E-03	1.15	97.24
56	N-Acetylneuraminic acid	$C_{11}H_{19}NO_9$	HMDB0000230	1.57	332.0949	[M+Na]+	0.22	4.73E-04	1.22	95.16
57	Citramalic acid	$C_5H_8O_5$	HMDB0000426	1.81	166.0727	[M+NH4]+	0.46	1.10E-03	1.18	95.16
58	1-Methyladenine	$C_6H_7N_5$	HMDB0011599	1.82	316.1751	[2M+NH4]+	0	9.34E-04	1.16	95.78
59	Glycerol 3-phosphate	$C_3H_9O_6P$	HMDB0000126	2.13	190.0495	[M+NH4]+	0.35	3.03E-05	1.33	97.2
60	L-Tryptophan	$C_{11}H_{12}N_2O_2$	HMDB0000929	2.13	203.0825	[M-H]-	0.18	1.44E-03	1.19	98.03
61	3-Hydroxyisovaleric acid	$C_5H_{10}O_3$	HMDB0000754	2.3	101.0595	[M+H-H2O]+	0.5	1.09E-04	1.27	98.3
62	Butyrylcarnitine	$C_{11}H_{21}NO_4$	HMDB0002013	2.46	232.1539	[M+H]+	0.08	1.71E-08	1.48	98.29
63	L-Glutamic acid	$C_5H_9NO_4$	HMDB0000148	2.63	189.0862	[M+ACN+H]+	0.25	2.87E-04	1.26	97.13
64	3-Methoxytyramine	$C_9H_{13}NO_2$	HMDB0000022	2.66	212.0923	[M+FA-H]-	0.44	2.03E-04	1.35	98.86
65	Biopterin	$C_9H_{11}N_5O_3$	HMDB0000468	2.7	272.0594	[M+Cl]-	0.24	4.66E-08	1.57	95.68
66	4-Guanidinobutanoic acid	$C_5H_{11}N_3O_2$	HMDB0003464	2.73	289.1651	[2M-H]-	2.42	2.52E-04	1.33	98.69
67	Acetylcysteine	$C_5H_9NO_3S$	HMDB0001890	2.77	162.0228	[M-H]-	0.51	3.52E-03	1.14	96.49
68	2-Keto-L-gluconate	$C_6H_{10}O_7$	HMDB0011732	2.77	193.0353	[M-H]-	0.28	3.26E-03	1.14	97.22
69	Tryptophol	$C_{10}H_{11}NO$	HMDB0003447	2.82	206.0817	[M+FA-H]-	11.44	6.29E-04	1.3	96.11
70	p-Cresol	C_7H_8O	HMDB0001858	2.83	107.0502	[M-H]-	0.42	3.85E-03	1.17	99.48
71	N-Acetyl-L-tyrosine	$C_{11}H_{13}NO_4$	HMDB0000866	2.89	204.0666	[M-H2O-H]-	7.37	2.08E-04	1.36	98
72	4-Hydroxybenzaldehyde	$C_7H_6O_2$	HMDB0011718	2.95	121.0293	[M-H]-	5.39	2.24E-03	1.21	97.08
73	N-Acetyl-D-glucosamine	$C_8H_{15}NO_6$	HMDB0000215	3	222.0987	[M+H]+	0.19	1.98E-03	1.14	96.95
74	Phenylpyruvic acid	$C_9H_8O_3$	HMDB0000205	3.03	206.0832	[M+ACN+H]+	2.99	1.19E-03	1.17	96.4

75	3-(3-Hydroxyphenyl)propanoic acid	$C_9H_{10}O_3$	HMDB0000375	3.03	131.0488	[M+H-2H2O]+	4.96	9.15E-05	1.3	98.32
76	Tyrosol	$C_8H_{10}O_2$	HMDB0004284	3.03	103.0538	[M+H-2H2O]+	3.7	2.76E-04	1.23	97.57
77	L-Isoleucine	$C_6H_{13}NO_2$	HMDB0000172	3.04	170.0603	[M+K]+	3.74	2.45E-04	1.25	98.6
78	Guanosine	$C_{10}H_{13}N_5O_5$	HMDB0000133	3.1	264.0738	[M-H2O-H]-	0.49	4.36E-03	1.14	98.97
79	Riboflavin	$C_{17}H_{20}N_4O_6$	HMDB0000244	3.22	377.1453	[M+H]+	4.32	6.70E-05	1.3	98.27
80	L-Lactic acid	$C_3H_6O_3$	HMDB0000190	3.28	179.0562	[2M-H]-	2.56	1.58E-03	1.22	96.07
81	20-Carboxy-leukotriene B4	$C_{20}H_{30}O_6$	HMDB0006059	3.46	411.203	[M+FA-H]-	3.87	7.99E-03	1.08	96.57
82	Phenylacetylglycine	$C_{10}H_{11}NO_3$	HMDB0000821	3.56	235.1078	[M+ACN+H]+	55.98	1.01E-03	1.18	97.89
83	Cortolone	$C_{21}H_{34}O_5$	HMDB0003128	3.7	365.2299	[M-H]-	2.97	1.88E-03	1.17	96.35
84	Dodecanoic acid	$C_{12}H_{24}O_2$	HMDB0000638	3.78	181.1594	[M-H2O-H]-	6.89	1.55E-05	1.45	98.06
85	Prostaglandin F2a	$C_{20}H_{34}O_5$	HMDB0001139	3.88	337.2434	[M+H-H2O]+	8.09	9.12E-05	1.31	96.67
86	L-Octanoylcarnitine	$C_{15}H_{29}NO_4$	HMDB0000791	4.11	288.2163	[M+H]+	0.13	1.26E-04	1.29	97.94
87	5-Methoxytryptamine	$C_{11}H_{14}N_2O$	HMDB0004095	4.15	379.2143	[2M-H]-	0.64	1.66E-02	1.02	96.29
88	Thymidine	$C_{10}H_{14}N_2O_5$	HMDB0000273	4.25	241.0851	[M-H]-	11.18	2.32E-04	1.34	96.64
89	Cholic acid	$C_{24}H_{40}O_5$	HMDB0000619	4.29	431.2755	[M+Na]+	35.41	8.81E-04	1.19	95.08
90	Chenodeoxycholic acid	$C_{24}H_{40}O_4$	HMDB0000518	4.39	437.2878	[M+FA-H]-	10.7	8.82E-04	1.24	95.17
91	Cortexolone	$C_{21}H_{30}O_4$	HMDB0000015	4.46	391.212	[M+FA-H]-	0.05	6.56E-10	1.62	96.09
92	N-Acetylaspartylglutamic acid	$C_{11}H_{16}N_2O_8$	HMDB0001067	4.78	285.0775	[M-H2O-H]-	12.7	2.49E-03	1.19	95.17
93	Decanoylcarnitine	$C_{17}H_{33}NO_4$	HMDB0000651	4.81	316.2478	[M+H]+	0.07	1.96E-04	1.26	97.21
94	Deoxycholic acid	$C_{24}H_{40}O_4$	HMDB0000626	5.1	391.2846	[M-H]-	67.61	1.13E-02	1	96.53
95	1-Methylhistamine	$C_6H_{11}N_3$	HMDB0000898	5.58	251.1978	[2M+H]+	0.58	3.11E-03	1.09	95.64
96	Palmitic acid	$C_{16}H_{32}O_2$	HMDB0000220	5.85	274.2734	[M+NH4]+	0.31	4.30E-04	1.2	97.12
97	Glucosamine	$C_6H_{13}NO_5$	HMDB0001514	6.3	221.1164	[M+ACN+H]+	0.57	1.42E-04	1.3	97.8
98	LysoPC(16:0/0:0)	$C_{24}H_{50}NO_7P$	HMDB0010382	6.35	496.3389	[M+H]+	0	4.70E-03	1.07	95.81
99	alpha-Linolenic acid	$C_{18}H_{30}O_2$	HMDB0001388	6.49	296.2577	[M+NH4]+	0.59	2.97E-03	1.08	97.93
100	Hydrocinnamic acid	$C_9H_{10}O_2$	HMDB0000764	6.82	301.1404	[2M+H]+	0.52	2.28E-03	1.12	97.56

101	Eicosapentaenoic acid	$C_{20}H_{30}O_2$	HMDB0001999	6.89	301.2164	[M-H]-	0.4	8.05E-03	1.05	98.2
102	Tetrahydrodeoxycorticosterone	$C_{21}H_{34}O_3$	HMDB0000879	6.98	333.2427	[M-H]-	0.59	3.74E-03	1.13	95.71
103	Myristic acid	$C_{14}H_{28}O_2$	HMDB0000806	6.99	227.2011	[M-H]-	0.5	5.42E-04	1.27	97.52
104	Palmitoleic acid	$C_{16}H_{30}O_2$	HMDB0003229	7.18	253.2168	[M-H]-	0.5	1.59E-04	1.38	96.74
105	Arachidonic acid	$C_{20}H_{32}O_2$	HMDB0001043	7.27	303.2324	[M-H]-	0.33	6.15E-03	1.08	96.79
106	LysoPC(18:0/0:0)	$C_{26}H_{54}NO_7P$	HMDB0010384	7.48	568.3604	[M+FA-H]-	0.02	1.51E-03	1.22	95.3
107	Oleic acid	$C_{18}H_{34}O_2$	HMDB0000207	7.8	281.2482	[M-H]-	0.5	1.96E-04	1.35	96.31
108	Tetrahydrocorticosterone	$C_{21}H_{34}O_4$	HMDB0000268	7.8	349.235	[M-H]-	0.73	8.55E-03	1.08	97.13
109	L-Palmitoylcarnitine	$C_{23}H_{45}NO_4$	HMDB0000222	8.27	797.651	[2M-H]-	0.39	1.77E-03	1.21	95.95

Table S3: The annotated information of biomarkers with reversal trend after CT extract treatment

No.	Compound	Formula	HMDB	RT(min)	M/Z	Adducts	Isotope Similarity	Con/AKI	AKI/HH
1	Glycerol 3-phosphate	$C_3H_9O_6P$	HMDB0000126	2.13	190.0495	[M+NH4]+	98.12	↓***	↑***
2	L-Glutamic acid	$C_5H_9NO_4$	HMDB0000148	2.63	189.0862	[M+ACN+H]+	98.12	↓***	↑**
3	Phenylpyruvic acid	$C_9H_8O_3$	HMDB0000205	3.03	206.0832	[M+ACN+H]+	95.95	↑**	↓*
4	Oleic acid	$C_{18}H_{34}O_2$	HMDB0000207	7.8	281.2482	[M-H]-	96.31	↓***	↑***
5	Palmitic acid	$C_{16}H_{32}O_2$	HMDB0000220	5.85	274.2734	[M+NH4]+	95.95	↓***	↑***
6	L-Palmitoylcarnitine	$C_{23}H_{45}NO_4$	HMDB0000222	8.27	797.651	[2M-H]-	96.28	↓**	↑***
7	Mevalonic acid	$C_6H_{12}O_4$	HMDB0000227	1.24	129.0555	[M-H2O-H]-	96.28	↑***	↓**
8	Tetrahydrocorticosterone	$C_{21}H_{34}O_4$	HMDB0000268	7.8	349.235	[M-H]-	95.95	↓**	↑***
9	Allantoin	$C_4H_6N_4O_3$	HMDB0000462	0.49	203.0153	[M+2Na-H]+	98.29	↓**	↑**
10	Galactonic acid	$C_6H_{12}O_7$	HMDB0000565	0.34	241.0289	[M+2Na-H]+	98.12	↓***	↑**
11	Hydrocinnamic acid	$C_9H_{10}O_2$	HMDB0000764	6.82	301.1404	[2M+H]+	98.12	↓**	↑***
12	N-Acetyl-L-aspartic acid	$C_6H_9NO_5$	HMDB0000812	0.32	176.0661	[M+H]+	96.28	↓***	↑***
13	Pimelic acid	$C_7H_{12}O_4$	HMDB0000857	0.69	319.1392	[2M-H]-	95.95	↑***	↓*
14	1-Methylhistamine	$C_6H_{11}N_3$	HMDB0000898	5.58	251.1978	[2M+H]+	95.64	↓**	↑***
15	Allysine	$C_6H_{11}NO_3$	HMDB0001263	0.79	184.0278	[M+K]+	98.29	↓**	↑*
16	Glucosamine	$C_6H_{13}NO_5$	HMDB0001514	6.3	221.1164	[M+ACN+H]+	98.12	↓***	↑***
17	Palmitoleic acid	$C_{16}H_{30}O_2$	HMDB0003229	7.18	253.2168	[M-H]-	95.95	↓***	↑***
18	Tryptophol	$C_{10}H_{11}NO$	HMDB0003447	2.82	206.0817	[M+FA-H]-	95.95	↑***	↓*
19	5-Methoxytryptamine	$C_{11}H_{14}N_2O$	HMDB0004095	4.15	379.2143	[2M-H]-	98.29	↓*	↑***
20	N2-Methylguanine	$C_6H_7N_5O$	HMDB0006040	1.33	166.0732	[M+H]+	96.28	↓***	↑***
21	3-Dehydroquinic acid	$C_7H_{10}O_6$	HMDB0012710	0.42	189.0403	[M-H]-	98.29	↓***	↑*

Table S4 Feature parameters of CT extract compound in the G-T-P network

No.	Name	Degree	Betweenness Centrality	Closeness Centrality
1	Quercetin	78	0.03880348	0.46494465
2	Apigenin	57	0.00385389	0.39252336
3	Luteolin	50	0.0000667	0.35897436
4	Kaempferol	42	0.02464443	0.43298969
5	Acacetin	40	0.20226369	0.64615385
6	Naringenin	31	0	0.32061069
7	Isoquercetin	16	0.03253203	0.46840149
8	Astragalin	16	0.04586767	0.49802372
9	Protocatechuic Acid	12	0.00435757	0.38297872
10	Taxifolin	12	0.00217907	0.37837838
11	Rutin	10	0.00310737	0.38297872
12	Kaempferol-3-O-Rutinoside	3	0.07950286	0.53164557
13	Hydroxysafflor Yellow A	1	0.00389139	0.39252336

Table S5 Characteristic parameters of target proteins in the G-T-P network (Top 10)

No.	Name	Degree	Betweenness Centrality	Closeness Centrality
1	RELA	22	0.00788266	0.516393
2	AKT1	20	0.00331421	0.480916
3	PIK3R1	19	0.00344017	0.492188
4	TP53	18	0.01513676	0.533898
5	MAPK14	18	0.00357803	0.492188
6	CDK2	18	0.01424302	0.508065
7	TNF	18	0.00405706	0.488372
8	GSK3B	17	0.00861215	0.520661
9	CASP3	17	0.0036885	0.492188
10	MAPK3	17	0.001537	0.473684

Table S6 Molecular docking results of compounds with disease targets

Ligand	Target	PDB ID	Affinity(kcal/mol)
Acacetin	AKT1	3O96	-9.5
	CASP3	3DEK	-7.8
	CDK2	1B38	-8.8
	GSK3B	1Q5K	-8.9
	MAPK14	2YIX	-10.0
	MAPK3	4QTB	-8.8
	PIK3R1	4L2Y	-8.5
	RELA	3RC0	-9.2
	TNF	2AZ5	-7.6
	TP53	3ZME	-8.0
Apigenin	AKT1	3O96	-9.6
	CASP3	3DEK	-7.9
	CDK2	1B38	-9.1
	GSK3B	1Q5K	-8.8
	MAPK14	2YIX	-9.7
	MAPK3	4QTB	-8.7
	PIK3R1	4L2Y	-8.6
	RELA	3RC0	-9.2
	TNF	2AZ5	-7.8
	TP53	3ZME	-7.9
Astragalin	AKT1	3O96	-10.2
	CASP3	3DEK	-8.0
	CDK2	1B38	-8.7
	GSK3B	1Q5K	-8.2
	MAPK14	2YIX	-8.6
	MAPK3	4QTB	-9.1
	PIK3R1	4L2Y	-8.8
	RELA	3RC0	-9.9
	TNF	2AZ5	-7.8
	TP53	3ZME	-7.9
Hydroxysafflor_yellow_a	AKT1	3O96	-9.4
	CASP3	3DEK	-8.5
	CDK2	1B38	-8.7
	GSK3B	1Q5K	-7.9
	MAPK14	2YIX	-6.5
	MAPK3	4QTB	-7.9
	PIK3R1	4L2Y	-7.3
	RELA	3RC0	-9.9
	TNF	2AZ5	-8.0
	TP53	3ZME	-7.6
Isoquercetin	AKT1	3O96	-10.0

	CASP3	3DEK	-8.3
	CDK2	1B38	-9.2
	GSK3B	1Q5K	-8.4
	MAPK14	2YIX	-7.7
	MAPK3	4QTB	-9.3
	PIK3R1	4L2Y	-7.7
	RELA	3RC0	-10.1
	TNF	2AZ5	-7.8
	TP53	3ZME	-7.3
Kaempferol	AKT1	3O96	-9.6
	CASP3	3DEK	-8.0
	CDK2	1B38	-9.4
	GSK3B	1Q5K	-8.8
	MAPK14	2YIX	-9.1
	MAPK3	4QTB	-8.6
	PIK3R1	4L2Y	-8.4
	RELA	3RC0	-9.2
	TNF	2AZ5	-7.2
	TP53	3ZME	-7.8
Kaempferol_3_O_Rutinoside	AKT1	3O96	-11.8
	CASP3	3DEK	-9.1
	CDK2	1B38	-10.5
	GSK3B	1Q5K	-9.2
	MAPK14	2YIX	-8.5
	MAPK3	4QTB	-9.2
	PIK3R1	4L2Y	-8.2
	RELA	3RC0	-9.9
	TNF	2AZ5	-8.5
	TP53	3ZME	-8.6
Luteolin	AKT1	3O96	-9.8
	CASP3	3DEK	-8.0
	CDK2	1B38	-9.4
	GSK3B	1Q5K	-8.8
	MAPK14	2YIX	-9.9
	MAPK3	4QTB	-8.8
	PIK3R1	4L2Y	-8.8
	RELA	3RC0	-9.1
	TNF	2AZ5	-7.9
	TP53	3ZME	-8.0
Naringenin	AKT1	3O96	-9.5
	CASP3	3DEK	-7.8
	CDK2	1B38	-8.6
	GSK3B	1Q5K	-8.7
	MAPK14	2YIX	-9.8

	MAPK3	4QTB	-8.5
	PIK3R1	4L2Y	-8.4
	RELA	3RC0	-8.8
	TNF	2AZ5	-7.7
	TP53	3ZME	-8.0
Protocatechuic_Acid	AKT1	3O96	-6.5
	CASP3	3DEK	-6.3
	CDK2	1B38	-5.9
	GSK3B	1Q5K	-5.4
	MAPK14	2YIX	-6.1
	MAPK3	4QTB	-6.1
	PIK3R1	4L2Y	-5.4
	RELA	3RC0	-6.2
	TNF	2AZ5	-5.6
	TP53	3ZME	-5.2
Quercetin	AKT1	3O96	-9.9
	CASP3	3DEK	-7.9
	CDK2	1B38	-9.7
	GSK3B	1Q5K	-8.5
	MAPK14	2YIX	-8.9
	MAPK3	4QTB	-8.6
	PIK3R1	4L2Y	-8.6
	RELA	3RC0	-9.2
	TNF	2AZ5	-7.9
	TP53	3ZME	-8.1
Rutin	AKT1	3O96	-11.6
	CASP3	3DEK	-9.0
	CDK2	1B38	-10.3
	GSK3B	1Q5K	-9.2
	MAPK14	2YIX	-7.0
	MAPK3	4QTB	-9.9
	PIK3R1	4L2Y	-8.8
	RELA	3RC0	-10.3
	TNF	2AZ5	-8.8
	TP53	3ZME	-7.9
Taxifolin	AKT1	3O96	-9.5
	CASP3	3DEK	-7.8
	CDK2	1B38	-9.3
	GSK3B	1Q5K	-8.5
	MAPK14	2YIX	-9.3
	MAPK3	4QTB	-8.7
	PIK3R1	4L2Y	-8.4
	RELA	3RC0	-8.8
	TNF	2AZ5	-7.9

	TP53	3ZME	-8.0
